# MicroRNA-1 protects the endothelium in acute lung injury

**DOI:** 10.1172/jci.insight.164816

**Published:** 2023-09-22

**Authors:** Asawari Korde, Maria Haslip, Prachi Pednekar, Alamzeb Khan, Maurizio Chioccioli, Sameet Mehta, Francesc Lopez-Giraldez, Santos Bermejo, Mauricio Rojas, Charles Dela Cruz, Michael A. Matthay, Jordan S. Pober, Richard W. Pierce, Shervin S. Takyar

**Affiliations:** 1 Department of Internal Medicine, Section of Pulmonary, Critical Care and Sleep Medicine, Yale University School of Medicine, New Haven, Connecticut, USA.; 2 Department of Medicine, Yale New Haven Hospital, New Haven, Connecticut, USA.; 3 Department of Immunobiology, and; 4 Department of Genetics, Yale University School Medicine, New Haven, Connecticut, USA.; 5 Division of Pulmonary, Critical Care and Sleep Medicine, The Ohio State University, Columbus, Ohio, USA.; 6 Cardiovascular Research Institute, Department of Medicine and Anesthesiology, UCSF, San Francisco, California, USA.

**Keywords:** Pulmonology, Vascular Biology, Endothelial cells, Noncoding RNAs

## Abstract

Acute lung injury (ALI) and its most severe form, acute respiratory distress syndrome (ARDS), cause severe endothelial dysfunction in the lung, and vascular endothelial growth factor (VEGF) is elevated in ARDS. We found that the levels of a VEGF-regulated microRNA, microRNA-1 (miR-1), were reduced in the lung endothelium after acute injury. Pulmonary endothelial cell–specific (EC-specific) overexpression of miR-1 protected the lung against cell death and barrier dysfunction in both murine and human models and increased the survival of mice after pneumonia-induced ALI. miR-1 had an intrinsic protective effect in pulmonary and other types of ECs; it inhibited apoptosis and necroptosis pathways and decreased capillary leak by protecting adherens and tight junctions. Comparative gene expression analysis and RISC recruitment assays identified miR-1 targets in the context of injury, including phosphodiesterase 5A (*PDE5A*), angiopoietin-2 (*ANGPT2*), CNKSR family member 3 (*CNKSR3*), and TNF-α–induced protein 2 (*TNFAIP2*). We validated miR-1–mediated regulation of ANGPT2 in both mouse and human ECs and found that in a 119-patient pneumonia cohort, miR-1 correlated inversely with ANGPT2. These findings illustrate a previously unknown role of miR-1 as a cytoprotective orchestrator of endothelial responses to acute injury with prognostic and therapeutic potential.

## Graphical abstract

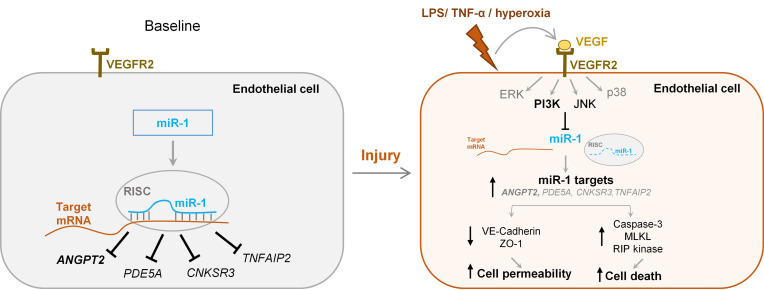

## Introduction

Acute lung injury (ALI) causes a progressive and widespread injury to the lung, leading to pulmonary edema, recruitment of inflammatory cells, and severe hypoxemia (1). Acute respiratory distress syndrome (ARDS), the most severe form of ALI, is a highly fatal disease with no available therapeutic agents. Lung endothelium is one of the main sites of cellular dysfunction in ARDS. Increased capillary leak in the early phase, and death of the endothelial cells (ECs) in the later stages, are ubiquitous features of ARDS (2). We and others have shown that gene expression is dysregulated in the endothelium of the acutely injured lung and alteration of this expression has substantial effects on the severity of ALI (3, 4). Several angiogenic mediators, including vascular endothelial growth factor (VEGF) and angiopoietin-2, are released during ALI and contribute to pulmonary endothelial dysfunction. In other contexts, VEGF functions as a growth and survival factor for ECs (5). To date, the fundamental mechanisms for VEGF-mediated lung injury are not completely understood.

MicroRNAs (miRNAs) are small noncoding RNAs that regulate gene expression by recruiting messenger RNAs (mRNAs) to the RNA-induced silencing complex (RISC) (6). miRNAs are deregulated in various disease states, including ALI, and impart cell- and context-specific effects on gene expression (7, 8). Therefore, it is often necessary to define the cellular site and context of a miRNA’s regulation to correctly identify its targets. A few miRNAs with specific roles in the injured lung endothelium have been described (9–12). miR-1 is one of the few VEGF-responsive miRNAs in the lung endothelium (13). VEGF treatment downregulates miR-1 expression and miR-1 overexpression inhibits VEGF-induced EC proliferation, suggesting that miR-1 serves as a negative regulator of VEGF angiogenic activity (14). However, unlike the majority of VEGF inhibitors that antagonize VEGF-mediated cell survival, miR-1 does not enhance EC apoptosis (14), suggesting that it is a unique VEGF blocker with possible endothelium-protective properties.

In this study, we first investigated the effects of ALI on miR-1 and its regulation. We then tested the importance of this regulation in cell-specific rescue experiments. In the second part of the manuscript, we used several cellular and molecular assays to define the mechanism of miR-1 effects in ALI. Finally, we performed an exploratory clinical study on a pneumonia cohort to test the potential translatability of our findings.

## Results

### Lung miR-1 levels are downregulated in ALI.

**Figure 1:**
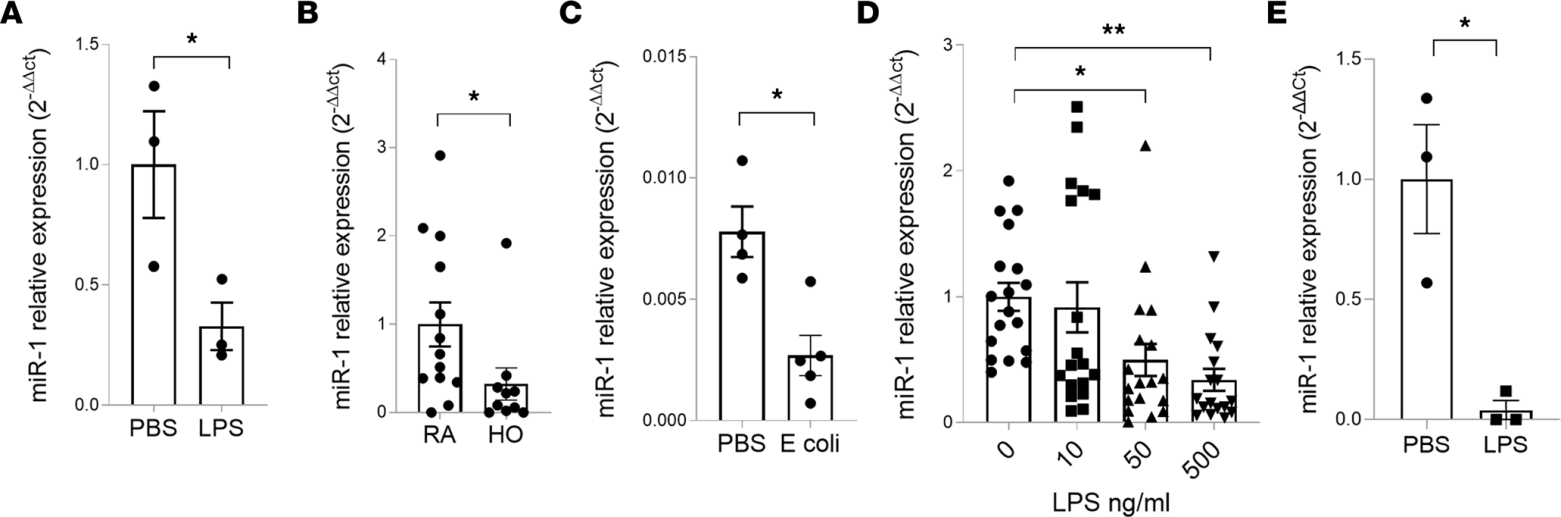
Lung injury downregulates miR-1. Mouse or human lung tissue were exposed to injury. Lung RNA was extracted and miR-1/18S levels were measured by qRT-PCR, analyzed by comparative Ct method, normalized to the control (PBS), and expressed as 2^–ΔΔCt^. (**A**) LPS model (*n* = 3). *P = 0.038. (**B**) Hyperoxia (HO) model (*n* = 10 in room air [RA] and 13 in HO). *P = 0.0201. (**C**) *E*. *coli* model (*n* ≥ 5). *P = 0.0062. (**D**) Human lung ex vivo culture: Human lung was exposed to various concentrations of LPS for 24 hours (*n* = 4 patients, 5 replicates each). *P = 0.0028, **P = 0.000025. (**E**) Ex vivo human lung perfusion: Human lungs were perfused with LPS (5 μg/kg ideal body weight of the donor) or PBS (control group) and biopsy samples were collected 6 hours after the initiation of perfusion (*n* = 3 donors). *P = 0.0478. Error bars represent the SEM. Data were analyzed by unpaired, 2-tailed *t* test with Welch’s correction or Mann-Whitney *U* test based on normality.

To test the effect of ALI on miR-1, we measured lung miR-1 levels in several murine ALI models, namely lipopolysaccharide (LPS) exposure, *E*. *coli* infection, and hyperoxia. All miR-1 measurements were performed using stem-loop quantitative RT-PCR that specifically quantifies the levels of the mature (the active form of) miR-1 (13). miR-1 levels were decreased in all the tested models (Figure 1, A–C). ALI experiments on cultured human lung tissue yielded similar results; LPS treatment in the culture media decreased human lung miR-1 levels in a dose-dependent manner (Figure 1D) and exposure to LPS (5 μg/kg ideal body weight of the donor) in the perfusate in an ex vivo lung perfusion experiment decreased miR-1 levels by 90%, compared with the controls (Figure 1E).

### miR-1 is downregulated in injured ECs.

**Figure 2:**
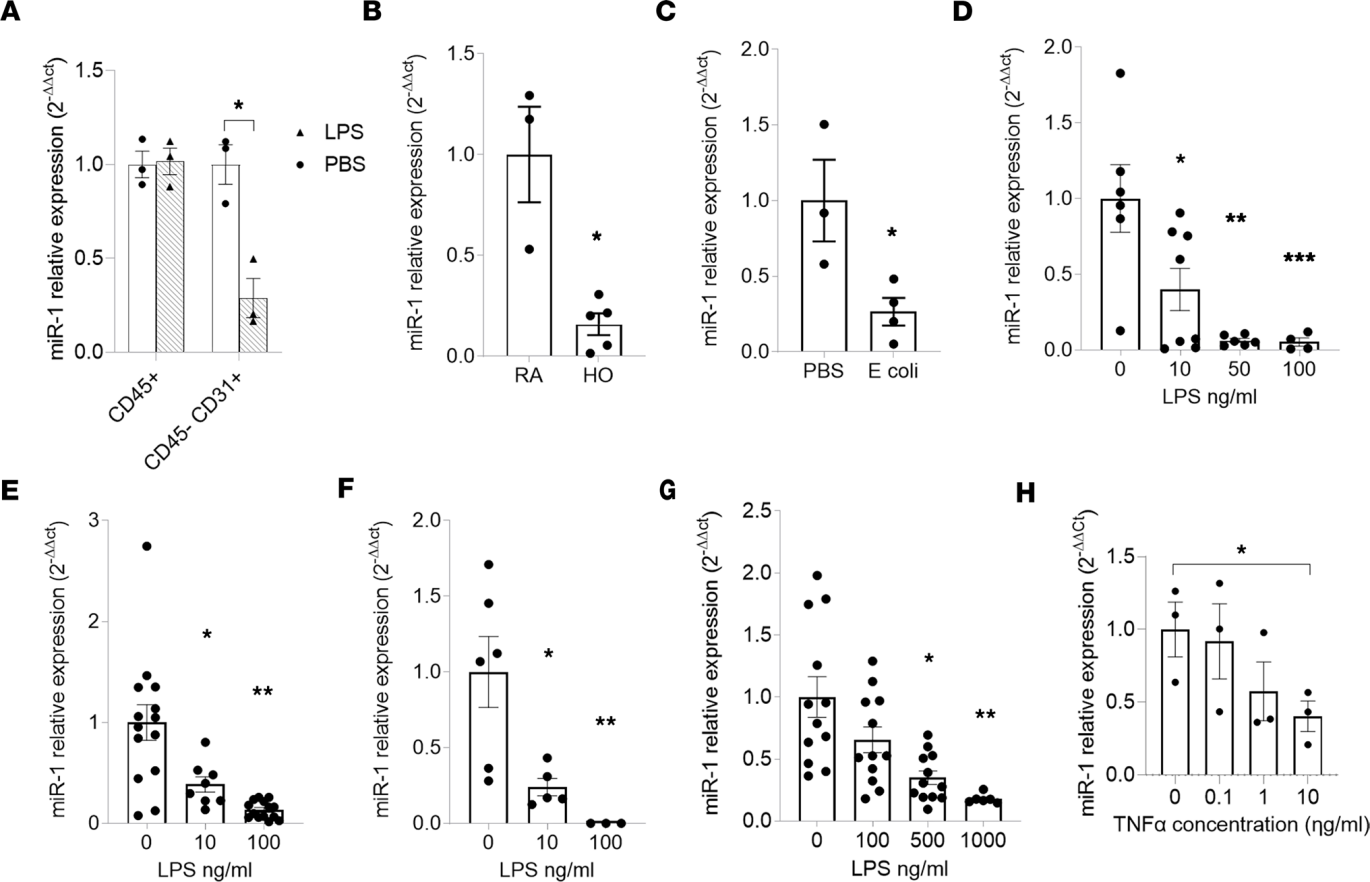
Lung injury downregulates miR-1 in endothelial cells. (**A**) Human ex vivo–cultured lungs were treated with LPS (500 ng/mL) or vehicle (PBS) for 24 hours. Endothelial (CD45^–^CD31^+^) and immune (CD45^+^) cells were isolated and miR-1 levels measured as described in Figure 1 (*n* = 3 patients). *P = 0.008. (**B**) Lung endothelial miR-1 in hyperoxia (HO) model; RA, room air (*n* ≥ 3). *P = 0.004. (**C**) Lung endothelial miR-1 in the *E*. *coli* model (*n* = 3 PBS, 4 for *E*. *coli*). *P = 0.0325. (**D**–**F**) Endothelial cells were treated with increasing concentrations of LPS for 24 hours and miR-1 levels were measured and expressed as described in **A**. (**D**) MLECs (*n* ≥ 4, from 2 experiments). *P = 0.0339, **P = 0.0019, ***P = 0.0097. (**E**) HPMECs (*n* ≥ 8 from 3 experiments). *P = 0.02, **P < 0.0001. (**F**) HUVECs (*n* = 6). *P = 0.02, **P = 0.008. (**G**) HDMECs (*n* = 12, from 2 experiments). *P = 0.0005, **P = 0.0001. (**H**) HPMECs were treated with increasing concentrations of TNF-α for 24 hours and mature miR-1 levels were measured, analyzed, and expressed as in **A** (*n* = 3). *P < 0.05. Error bars represent the SEM. Data were analyzed by unpaired, 2-tailed *t* test with Welch’s correction or Mann-Whitney *U* test based on normality.

Since VEGF increases in ALI and VEGF overexpression specifically decreases miR-1 levels in the lung endothelium (13–15), we asked whether the observed downregulation of miR-1 in the injured lung occurs specifically in the endothelial compartment. We measured mature miR-1 levels in the immune cells (CD45^+^) and ECs (CD45^–^CD31^+^) isolated from LPS-treated (or vehicle-treated) human lungs and found that miR-1 was only downregulated in the EC population (Figure 2A). Measurements of miR-1 in the lung ECs in the hyperoxia (Figure 2B) and *E*. *coli* infection (Figure 2C) models yielded similar results. We next tested whether this downregulation could be modeled in cultured ECs. As shown in Figure 2, D and E, LPS treatment led to dose-dependent miR-1 reductions in both mouse and human pulmonary microvascular ECs (MLECs and HPMECs, respectively). Interestingly, LPS had a similar effect on other EC types. Treatment of 2 unrelated ECs, human umbilical vein ECs (HUVECs) and human dermal microvascular ECs (HDMECs), led to similar dose-dependent miR-1 downregulation (Figure 2, F and G). We also tested the effect of tumor necrosis factor α (TNF-α), one of the main mediators of cellular injury in ALI (16), on endothelial miR-1. Treatment with TNF-α, similar to LPS, downregulated mature miR-1 in HPMECs in a dose-dependent manner (Figure 2H). These observations strongly suggest that miR-1 downregulation is a universal endothelial response to injury and is not limited to pulmonary infections.

### VEGF drives miR-1 downregulation in the injured endothelium.

**Figure 3:**
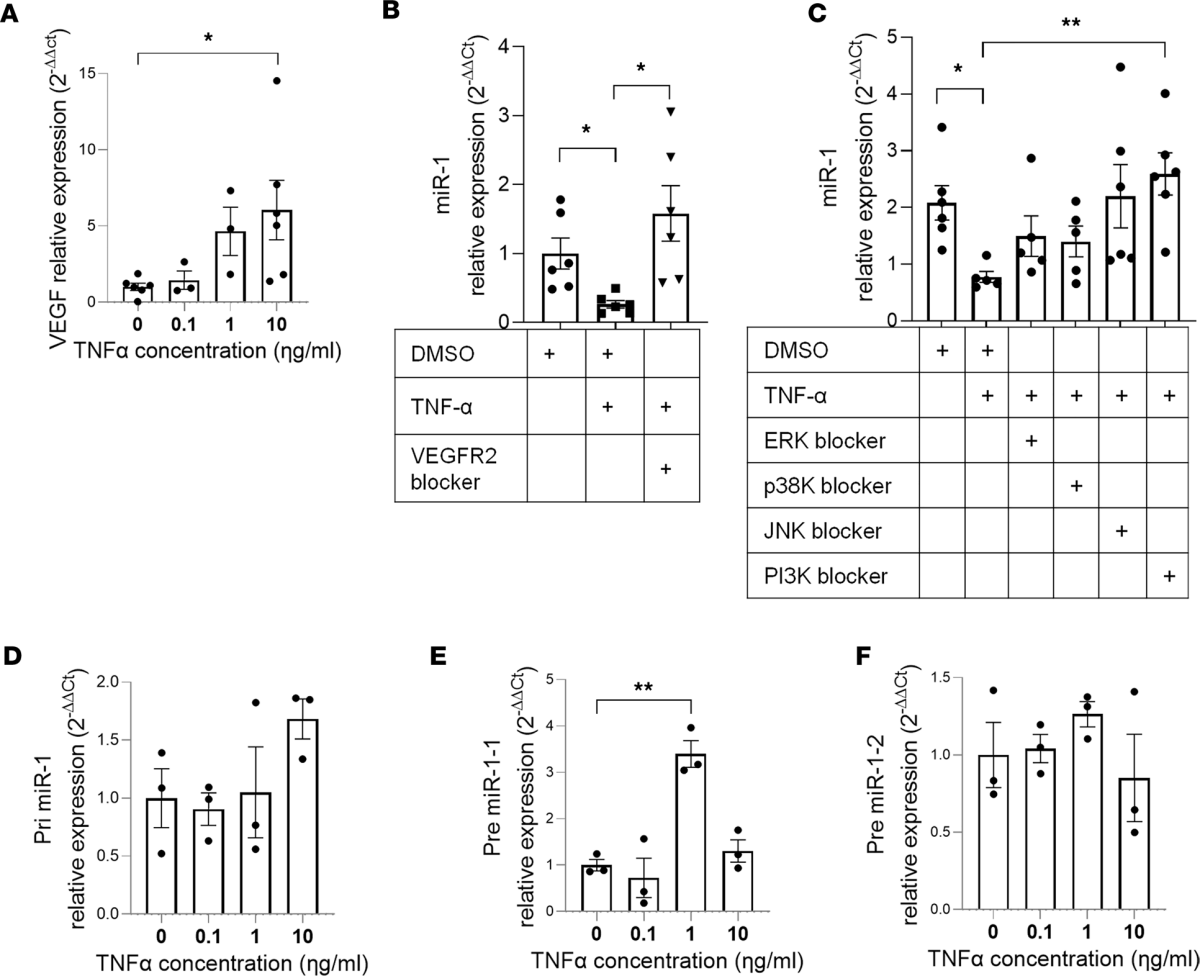
The mechanism of miR-1 downregulation in lung injury. (**A**) HPMECs were treated with increasing concentrations of TNF-α for 24 hours. VEGF/18S mRNA levels were measured by qRT-PCR, analyzed by the comparative Ct method, and normalized values expressed as 2^–ΔΔCt^ (*n* > 3, from 2 experiments). *P = 0.04. (**B**) HPMECs were incubated with VEGFR2 blocker (SU5614) for 2 hours followed by TNF-α treatment for 24 hours. miR-1/18S levels were measured and expressed as in Figure 2A (*n* = 6). *P < 0.022. (**C**) HPMECs were incubated with various inhibitors followed by TNF-α treatment. miR-1/18S levels were measured and expressed as in Figure 2A (*n* > 5). *P = 0.006, **P = 0.003. (**D**–**F**) HPMECs were treated with increasing concentrations of TNF-α for 24 hours. The levels of pre- and pri-miR-1/18s transcripts were measured by qRT-PCR, analyzed by comparative Ct method, and normalized values expressed as 2^–ΔΔCt^. (**D**) pri-miR-1 (*n* = 3), (**E**) pre-miR-1-1 (*n* = 3, **P = 0.006), and (**F**) pre-miR-1-2 (*n* = 3). Error bars represent the SEM. Data were analyzed by unpaired, 2-tailed *t* test with Welch’s correction or Mann-Whitney *U* test based on normality. ERK, extracellular signal–regulated kinase; P38K, p38 mitogen–activated protein kinase; JNK, c-jun N-terminal kinase; PI3K, phosphoinositide 3-kinase; VEGFR2, vascular endothelial growth factor receptor 2; Pri-, primary miR-1; Pre, precursor miR-1 transcript.

We next focused on the upstream mechanisms that regulate miR-1. miR-1 is a VEGF-responsive miRNA and VEGF is one of the main mediators of vascular dysfunction in ALI (13). Therefore, we tested the status of VEGF and its effect on miR-1 in the injured endothelium. Consistent with previous reports (17, 18), treatment with TNF-α or LPS increased VEGF expression in HPMECs (Figure 3A and Supplemental Figure 1; supplemental material available online with this article; https://doi.org/10.1172/jci.insight.164816DS1). More importantly, the blockade of VEGF receptor 2 (VEGFR2), the main receptor for VEGF in ECs, completely abrogated miR-1 downregulation, showing the central role of VEGF signaling in miR-1 regulation (Figure 3B). Following this line of inquiry, we tested the contribution of VEGF-downstream pathways in blockade experiments. The blockade of PI3 kinase (and JNK, although not statistically significant) caused the most marked increase in miR-1 levels and p38 or ERK blockade had only partial effects (Figure 3C). Taken together, these experiments show that VEGF signaling through PI3 kinase, and possibly other downstream pathways, is the main driver of miR-1 downregulation in the acutely injured endothelium.

### Injury specifically regulates mature miR-1.

We next examined the mechanism by which acute injury reduces miR-1. We modeled this process by adding TNF-α to cultured human umbilical vein ECs. Since miRNAs are generated through sequential enzymatic steps that produce primary (pri-), precursor (pre-), and finally the mature miRNA, which is the active form of the miRNA gene, we measured the levels of these sequences in the injured ECs. As shown above (Figure 2H), TNF-α downregulates mature miR-1 in a dose-dependent manner. In contrast, pri-miR-1 levels were increased at the highest TNF-α levels tested and the pre-miR-1-1 and pre-miR-1-2 levels did not show any specific dose-response pattern (Figure 3, D–F). These results suggest that injury reduces the level of the mature miRNA without reducing the levels of its precursors.

### EC-specific miR-1 protects the lung against ALI.

**Figure 4:**
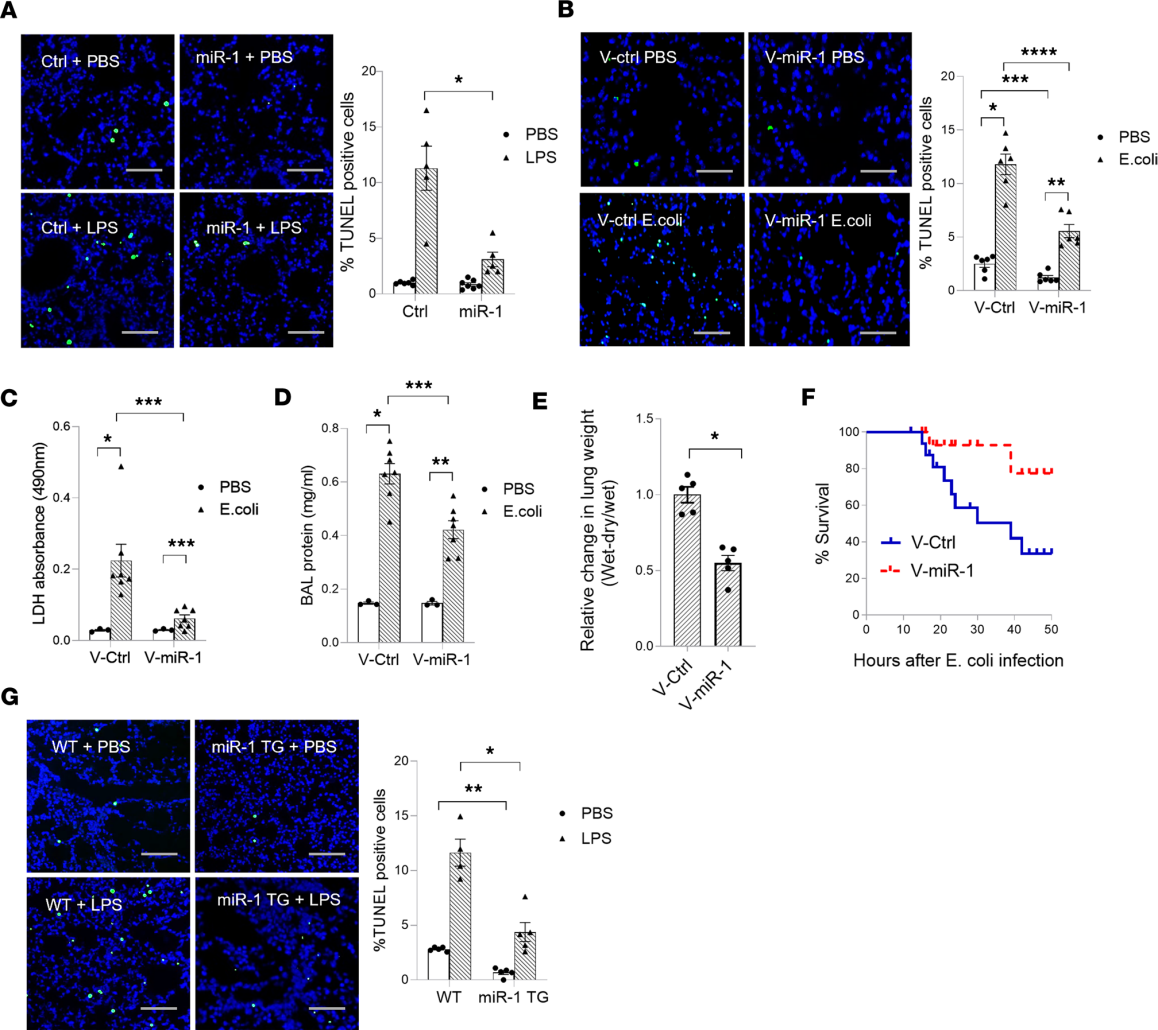
Endothelial miR-1 protects murine lung from injury. (**A**) Mice received a double-stranded miR-1 RNA mimic (miR-1) or control RNA (ctrl) intranasally, followed by intranasal LPS (4 mg/kg). TUNEL assay was performed on the lung tissues and percentage TUNEL-positive/total (DAPI-positive) cells was calculated and graphed. Images were acquired at ×200 magnification (*n* > 4 for PBS groups, *n* = 5 for LPS groups). *P = 0.0159. (**B**–**F**) Mice received endothelial cell–specific lentiviral vector V-miR-1 or control (V-ctrl) and were infected with *E*. *coli* through the intranasal route. Lungs were harvested 24 hours after infection and processed for various indices. (**B**) Percentage TUNEL-positive cells was determined and graphed as in **A** (*n* = 3 for PBS, 7 for *E*. *coli*). *P = 0.00004, **P = 0.00027, ***P = 0.00566, ****P = 0.00025. (**C**) LDH was measured in the BAL collected from murine lungs (*n* = 3 PBS and *n* = 7 *E*. *coli*). *P = 0.005364, **P = 0.011498, ***P = 0.018881. (**D**) Total protein in BAL was measured using BCA assay (*n* = 3 for PBS and 7 for *E*. *coli*). *P = 0.00000631, **P = 0.0000677, ***P = 0.0000745. (**E**) Lung water was measured and expressed as relative weight change ([wet – dry]/wet weight, *n* = 5). *P = 0.000502. (**F**) Kaplan-Meier curves for the survival of mice. Difference between the groups was analyzed using log-rank (Mantel-Cox) test (*n* = 17 per group, from 2 experiments). *P = 0.033. (**G**) miR-1–transgenic (miR-1 TG) and WT mice were exposed to LPS (4 mg/kg) or control and percentage TUNEL-positive cells was determined as in **A** (*n* ≥ 4). *P = 0.015, **P = 0.0079. Scale bars: 100 μm. Error bars represent the SEM. Data were analyzed by unpaired, 2-tailed *t* test with Welch’s correction or Mann-Whitney *U* test based on normality.

To evaluate the importance of mature miR-1 downregulation in ALI, we tested the effects of its reversal (i.e., increasing miR-1 levels) on ALI severity. We started these experiments with direct delivery of mature miR-1. We had previously reported that intranasal delivery of a double-stranded mature miR-1 mimic leads to high levels of miR-1 in the lung endothelium (13). Direct miR-1 delivery (compared with scrambled control) decreased LPS-induced lung cell death by approximately 70% (Figure 4A). This suggested that low miR-1 levels are likely pathogenic in severe ALI and that increasing miR-1 levels has a protective effect. Since miR-1 was specifically downregulated in the endothelium, we asked whether isolated overexpression of miR-1 in the endothelium would also be protective. We used a vascular-specific lentiviral vector (V-miR-1) (14, 15) in the *E*. *coli* infection model and measured indices of ALI severity in the lung and bronchoalveolar lavage (BAL). V-miR-1 (compared with the control vector) reduced lung cell death (measured by TUNEL staining and LDH release; Figure 4, B and C, respectively), and permeability and lung pulmonary edema (measured by BAL protein and lung water content; Figure 4, D and E, respectively), and increased the survival of the injured mice (Figure 4F), without significantly affecting the macrophage or neutrophil counts in the BAL (Supplemental Figure 2). V-miR-1 had a similarly protective effect in the hyperoxia model (Supplemental Figure 3), suggesting that its effect is not limited to the infectious models. To test whether the route and method of miR-1 overexpression affect its properties, we used our EC-specific miR-1–transgenic mice (14, 15). Induction of the miR-1 transgene in this model had a protective effect similar to V-miR-1 delivery (Figure 4G and Supplemental Figure 4). Also, similar to the lentiviral model, miR-1 transgenic expression did not affect leucocyte counts in BAL or murine lungs (in the alveolar, peribronchial, or interstitial areas; Supplemental Figure 5).

### miR-1 is protective in the human lung.

**Figure 5:**
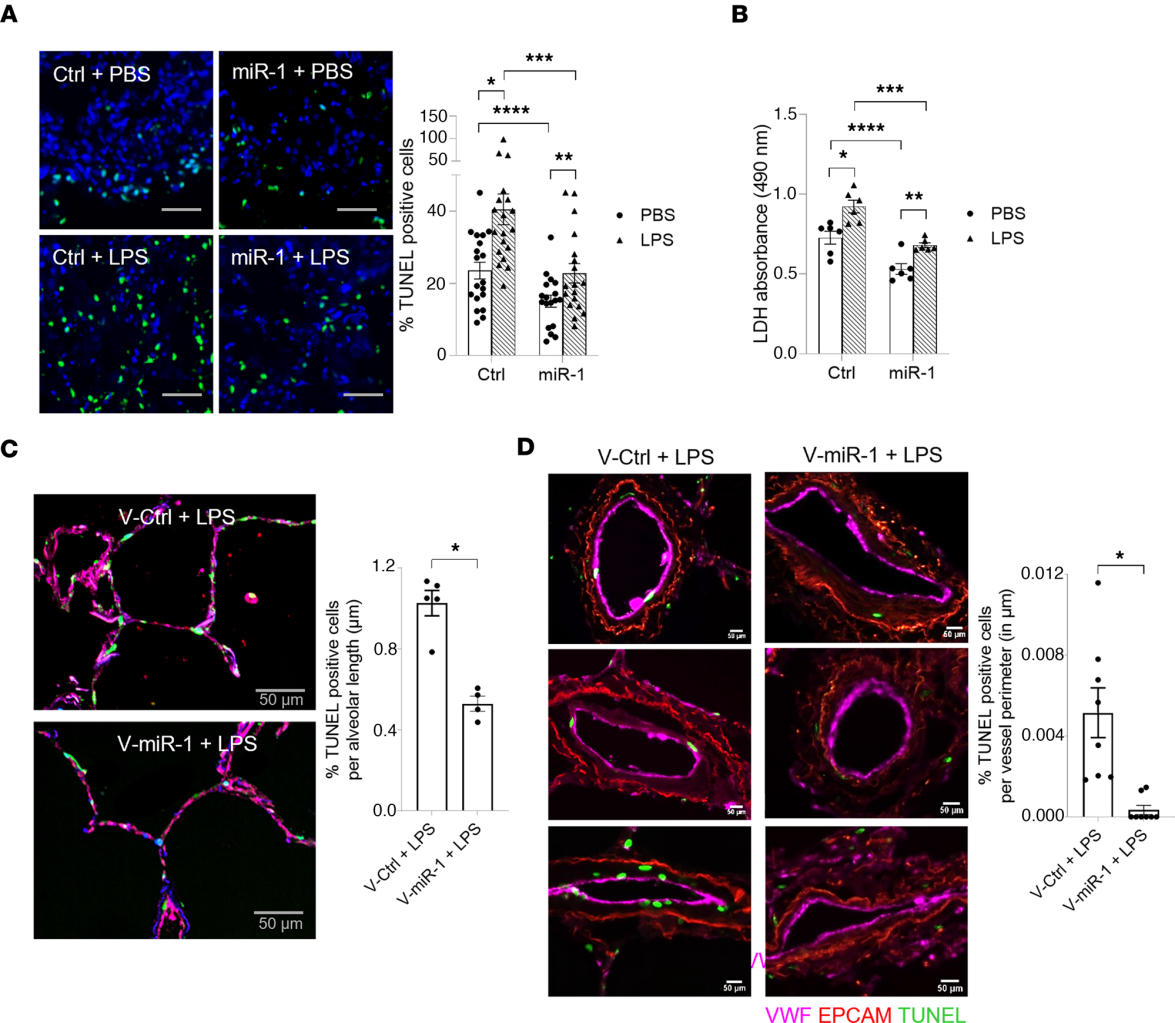
miR-1 protects human lungs against cell death. Human lung tissue samples were cultured and transfected with a double-stranded miR-1 RNA mimic (miR-1) or control RNA (ctrl), followed by 24-hour treatment with LPS (500 ng/mL) or vehicle (PBS). (**A**) Percentage TUNEL-positive cells was measured and graphed as in Figure 4A (*n* = 3 patients, 6 replicates each). *P = 0.0016, **P = 0.0225, ***P = 0.0014, ****P = 0.0058. Scale bars: 100 μm. (**B**) LDH levels were measured in culture media, and the values were normalized to the mean of the PBS group (*n* = 3 patients, 2 replicates each). *P = 0.0067, **P = 0.0052, ***P = 0.0011, ****P = 0.0043. (**C** and **D**) PCLS samples from the human lung were cultured in the growth medium, transduced with V-miR-1 or V-Ctrl, and treated with LPS (500 ng/mL) for 24 hours. Paraffin-embedded sections were stained for TUNEL, von Willebrand factor (VWF, for endothelial cells), and epithelial cell adhesion molecule (EpCAM, for epithelial cells). (**C**) Representative confocal images (×200 magnification) of alveoli. The graph represents the quantification of percentage TUNEL-positive cells/length of the alveolar wall (*n* = 5 replicates for V-ctrl and 4 for V-miR-1). *P = 0.0004. (**D**) Representative images of small lung vessels. The graph represents percentage TUNEL-positive cells/perimeter of the vessel (*n* = 6 replicates per group). Scale bars: 50 μm (**C** and **D**). *P = 0.0002. Error bars represent the SEM. Data were analyzed by unpaired, 2-tailed *t* test with Welch’s correction or Mann-Whitney *U* test based on normality.

In the next set of experiments, we tested the relevance of our findings in human lungs. We first tested the effect of miR-1 on lung cell death in ex vivo organ culture. miR-1–transfected (and control-transfected) lungs were treated with LPS (or vehicle) and cell death was assessed by TUNEL assay and LDH release. miR-1 transfection was protective and decreased both cell death (Figure 5A) and LDH release (Figure 5B) by 30% and 50%, respectively, with and without LPS treatment. We repeated this experiment by applying a V-miR-1 vector to a precision-cut lung slice (PCLS) preparation to test the effect of vascular-specific miR-1 and delineate the compartment(s) protected by it. We also costained the lungs for a vascular marker (von Willebrand factor, VWF) to specifically assess the status of the lung ECs. As shown in Figure 5, C and D, the EC-specific miR-1 decreased cell death in the alveolar compartment, the main site of injury in ARDS. Extra-alveolar vasculature, which includes larger (diameter > 25 μm) extraparenchymal vessels (19), was also protected. These results strongly suggest that miR-1 directly protects the ECs within the lung, including the alveolar vessels.

### miR-1 protects ECs through an intrinsic mechanism.

To confirm that miR-1 has an intrinsic protective effect in ECs, i.e., it protects the ECs in which it is expressed, we measured cell death in flow-sorted lung ECs from our miR-1–transgenic mice and their control littermates. Similar to the human lung, cell-specific overexpression of miR-1 in the endothelium decreased EC cell death in the lungs (Supplemental Figure 6), confirming the intrinsic effect of miR-1. We further investigated these properties in cell culture. We again used TNF-α to model acute endothelial injury. TNF signaling through its primary receptor, TNFR1, occurs in 2 phases mediated by different signaling complexes. Initially, ligand binding to TNFR1 induces new gene expression that disrupts the barrier formed by EC monolayers, an effect that peaks at around 6 hours. Subsequently, TNFR1 recruits and activates proteins that initiate apoptosis and or necroptosis, inducing measurable cell death within 24 to 48 hours (20).

**Figure 6:**
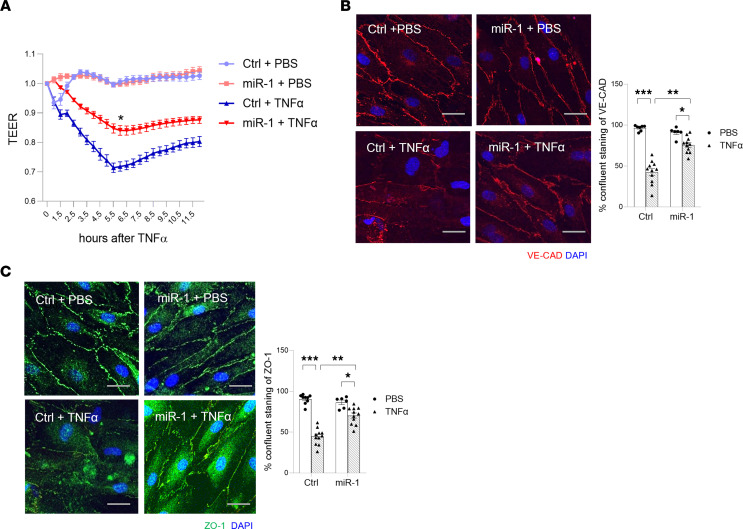
miR-1 reduces endothelial cell permeability. HPMECs were transfected with miR-1 or control RNA (Ctrl) and treated with TNF-α (10 ng/mL) or vehicle (PBS). (**A**) The permeability (transendothelial electric resistances, TEER) of the endothelial monolayers was measured at the given time points and plotted after normalization to the baseline values (*n* = 24, from 2 experiments). *P = 0.0001 at 6 hours. (**B** and **C**) Cells were stained for VE-cadherin (red) or zonula occludens-1 (ZO-1, green) and with DAPI (nuclei in blue). Images were collected from randomly selected fields and percentage confluently stained/total stained cell circumference was quantified by fluorescence microscopy. Values were normalized to the control/PBS group. (**B**) Representative images (left panel) and graphs (right panel) for VE-cadherin. (**C**) Representative images (left panel) and graphs (right panel) for ZO-1 (*n* ≥ 5 per group). Scale bars: 25 μm. *P < 0.01, **P < 0.001, ***P < 0.0001. Error bars represent the SEM. Data were analyzed by unpaired, 2-tailed *t* test with Welch’s correction or Mann-Whitney *U* test based on normality.

To study the miR-1–mediated protection against barrier dysfunction, we tested the effect of miR-1 transfection on transendothelial electric resistance (TEER) of confluent HPMEC monolayers, measured by electrical cell-substrate impedance sensing (ECIS). As shown in Figure 6A, miR-1 reduced the magnitude of the TNF-α–induced loss of TEER by approximately 50% at 6 hours, compared with the scrambled control. In accord with this finding, immunofluorescence microscopy (Figure 6, B and C) showed relative preservation of the VE-cadherin and zonula occludens-1 (ZO-1) staining in miR-1–transfected cells, compared with controls, suggesting that miR-1 has a direct effect on the endothelial adherens and tight junctions.

**Figure 7:**
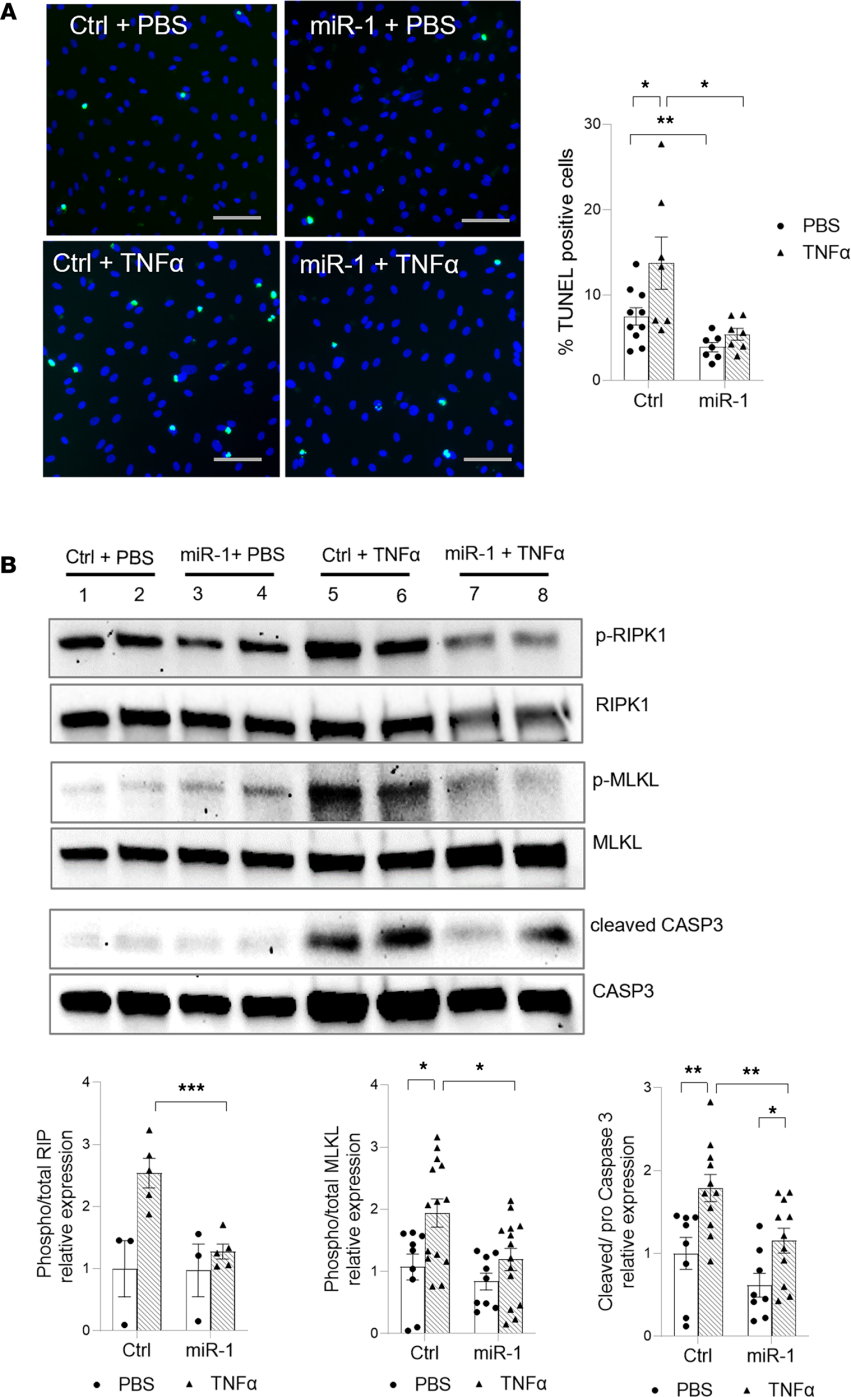
miR-1 protects endothelium from cell death. HPMECs were transfected with miR-1 or control (Ctrl) RNA and treated with TNF-α (10 ng/mL) or vehicle (PBS) for 24 hours. (**A**) Percentage TUNEL-positive cells was measured and graphed as in Figure 4A. Images were acquired under ×40 magnification (*n* ≥ 7 per group). Scale bars: 50 μm. *P = 0.0063, **P = 0.022. (**B**) Phosphorylated and total RIPK1 and MLKL proteins and cleaved and total caspase 3 (CASP3) were detected by Western blotting. The panel on the left shows representative immunoblots and the panel on the right shows quantification for each group normalized to the control/PBS group (*n* ≥ 3 for RIPK1 and *n* ≥ 9 for MLKL and CASP3). *P < 0.05, **P < 0.01, ***P < 0.005. Error bars represent the SEM. Data were analyzed by unpaired, 2-tailed *t* test with Welch’s correction or Mann-Whitney *U* test based on normality. RIPK1, receptor-interacting protein kinase 1; MLKL, mixed-lineage kinase domain–like pseudokinase.

In cell death experiments, miR-1 transfection, compared with the scrambled control, decreased cell death in HPMECs and HUVECs by more than 50%, both at baseline and after 24 hours of TNF-α treatment (Figure 7A and Supplemental Figure 7). To gain insight into the mechanism of this protection, we examined the activation status of programmed cell death pathways. miR-1 (compared with the scrambled control) significantly reduced the activation of receptor-interacting serine/threonine kinase 1 (RIPK1), and mixed-lineage kinase domain–like pseudokinase (MLKL) enzymes, and cleavage of caspase 3 after TNF-α exposure but did not have a significant effect on gasdermin D cleavage (Figure 7B and Supplemental Figure 8), suggesting that it mainly targets necroptosis and apoptosis, but not the pyroptosis pathway. These observations confirmed the intrinsic effect of miR-1 in the ECs and strongly suggested that it directly targets an endothelial gene network.

### miR-1 targets injury-related genes in the endothelium.

**Figure 8:**
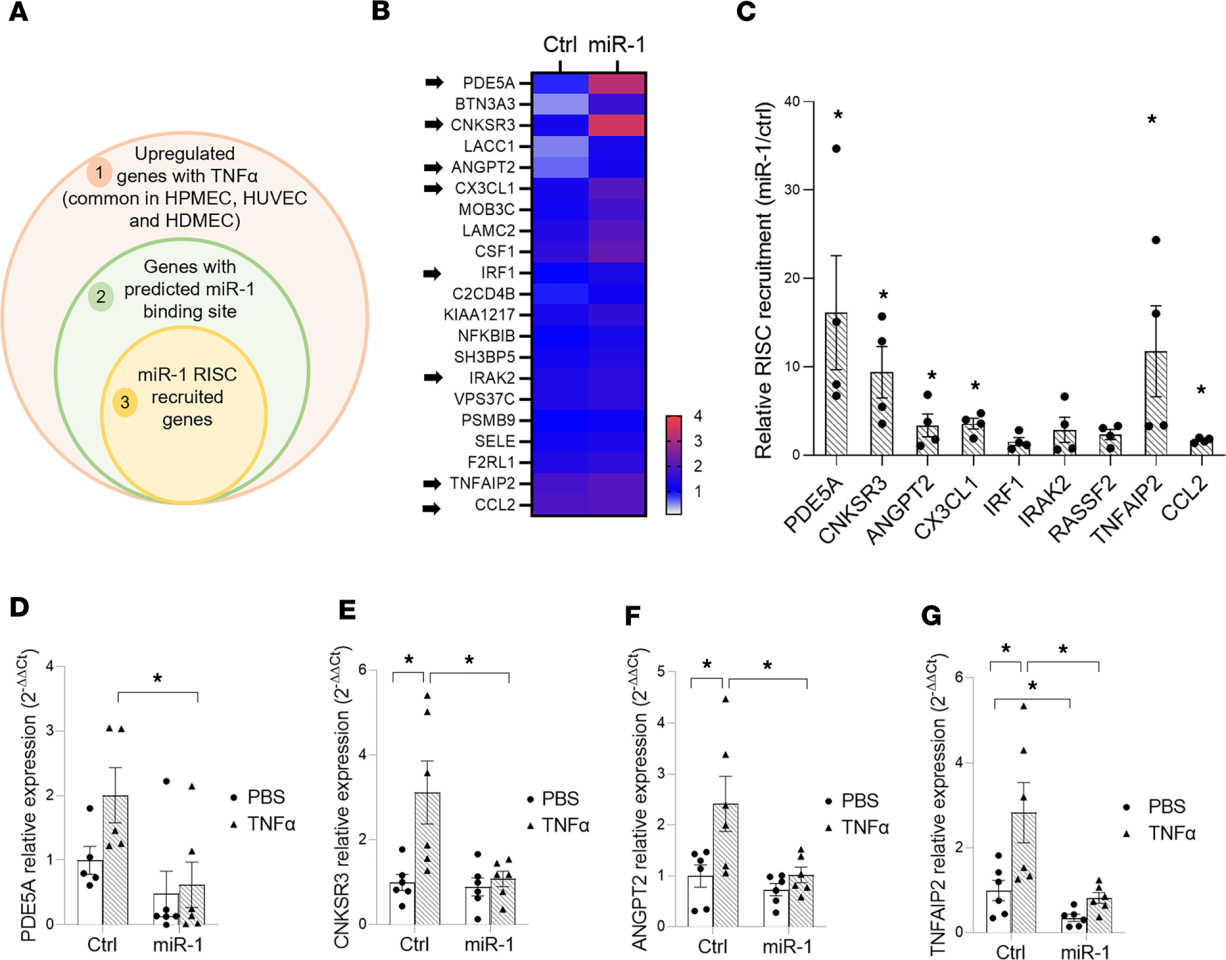
miR-1 targets in the injured endothelium. (**A**) Schematic representation of our strategy for selecting the candidate genes. Circles represent the genes that were selected based on the criteria in each group. (**B**) Heatmap shows the RISC-recruited genes (circle 3 in **A**). Arrows show the genes from the list that were known to be involved in ALI or cell death. (**C**) Validation of RISC recruitment for the selected genes (marked by arrows in **B**). HUVECs were transduced with V-miR-1 or control (V-ctrl) and their lysates were used in an Ago pull-down assay. Expression levels for the selected genes were measured by qRT-PCR in the Ago-immunoprecipitated fraction and normalized to their expression levels in the whole lysate. The values for V-miR-1–transduced cells /V-ctrl–transduced cells are expressed as relative RISC recruitment (*n* = 4, from 2 experiments). *P < 0.05. (**D**–**G**) HUVECs were transfected with miR-1 or control RNA (Ctrl) and treated with TNF-α (10 ng/mL) or vehicle (PBS) and levels of each mRNA/18S in the lysates were measured and expressed as in Figure 3A. Graphs show the levels for (**D**) phosphodiesterase 5A (*PDE5A*), (**E**) connector enhancer of kinase suppressor of Ras (*CNKSR3*), (**F**) angiopoietin-2 (*ANGPT2*), and (**G**) TNF-α–induced protein 2 (*TNFAIP*) (*n* = 6 per group in each, from 2 experiments). *P < 0.05. Error bars represent the SEM. Data were analyzed by unpaired, 2-tailed *t* test with Welch’s correction or Mann-Whitney *U* test based on normality.

To identify key targets of miR-1 in acutely injured endothelium, we made use of the ubiquity of the miR-1 response in different ECs and the molecular mechanism of miRNA-mediated regulation, i.e., recognition of a response element in the 3′ untranslated region (3′UTR) of their target mRNAs and recruiting them to the RISC. We used 3 sequential analytic steps to identify these specific targets: a comparative gene expression analysis on 3 different types of ECs before and after injury, miRNA target prediction based on 4 different algorithms, and a RISC recruitment (Argonaute pull down) assay on miR-1–transfected cells (Figure 8A). Since miRNAs inhibit the expression of their targets and miR-1 is downregulated in the injured endothelium, miR-1 target mRNAs are expected to (a) be upregulated in response to injury, (b) have a miR-1 binding site, and (c) get recruited to RISC by miR-1. We performed RNA sequencing on HUVECs, HPMECs, and HDMECs treated with TNF-α (or control) to find the common genes that are increased after injury (>0.5 log[fold change] with TNF-α, *P* < 0.05) in all 3 types of human ECs (Supplemental Table 1, 161 genes). We then used 4 established prediction algorithms, miRcode, miRWalk, miRDB, and miRTarBase, to identify the genes in this group that contained a miR-1 binding site in their 3′UTR (Supplemental Table 2, 64 genes). Finally, we did an Argonaute pull-down assay followed by high-throughput RNA sequencing (Ago-seq) on miR-1–transfected ECs (versus controls) to identify all the mRNAs that were recruited to the RISC by miR-1. A comparison of these genes with our 64-gene candidate list yielded a final list of 21 miR-1 targets in the injured endothelium (Figure 8B, 21 genes).

Several of these genes, including phosphodiesterase 5A (*PDE5A*), CNKSR family member 3 (*CNKSR3*), angiopoietin-2 (*ANGPT2*), C-X3-C motif chemokine ligand 1 (*CX3CL1*), interferon regulatory factor 1 (*IRF1*), IL-1 receptor–associated kinase 2 (*IRAK2*), Ras association domain family member 2 (*RASSF2*), TNF-α–induced protein 2 (*TNFAIP2*), and c-c motif chemokine ligand-2 (*CCL2*) are known to play critical roles in cell death and/or ALI (21–26) and their targeting by miR-1 is consistent with the miR-1 protective effects. We did further testing to validate the targeting of these genes. Gene-specific qRT-PCR on the input (whole lysate) and Ago-bound fractions from our Argonaute pull-down assay confirmed the enrichment of *PDE5A*, *CNKSR3*, *ANGPT2*, *CX3CL1*, *TNFAIP2*, and *CCL2* genes in the Ago-bound fraction (Figure 8C). To investigate further, we measured the levels of these genes in TNF-α–injured endothelium and the effect of miR-1 on their expression (Figure 8, D–G). TNF-α treatment increased, and miR-1 transfection decreased, the expression of all of these genes in ECs, confirming their targeting by miR-1 in the context of injury.

### ANGPT2 is one of the mediators of the miR-1 protective effect.

**Figure 9:**
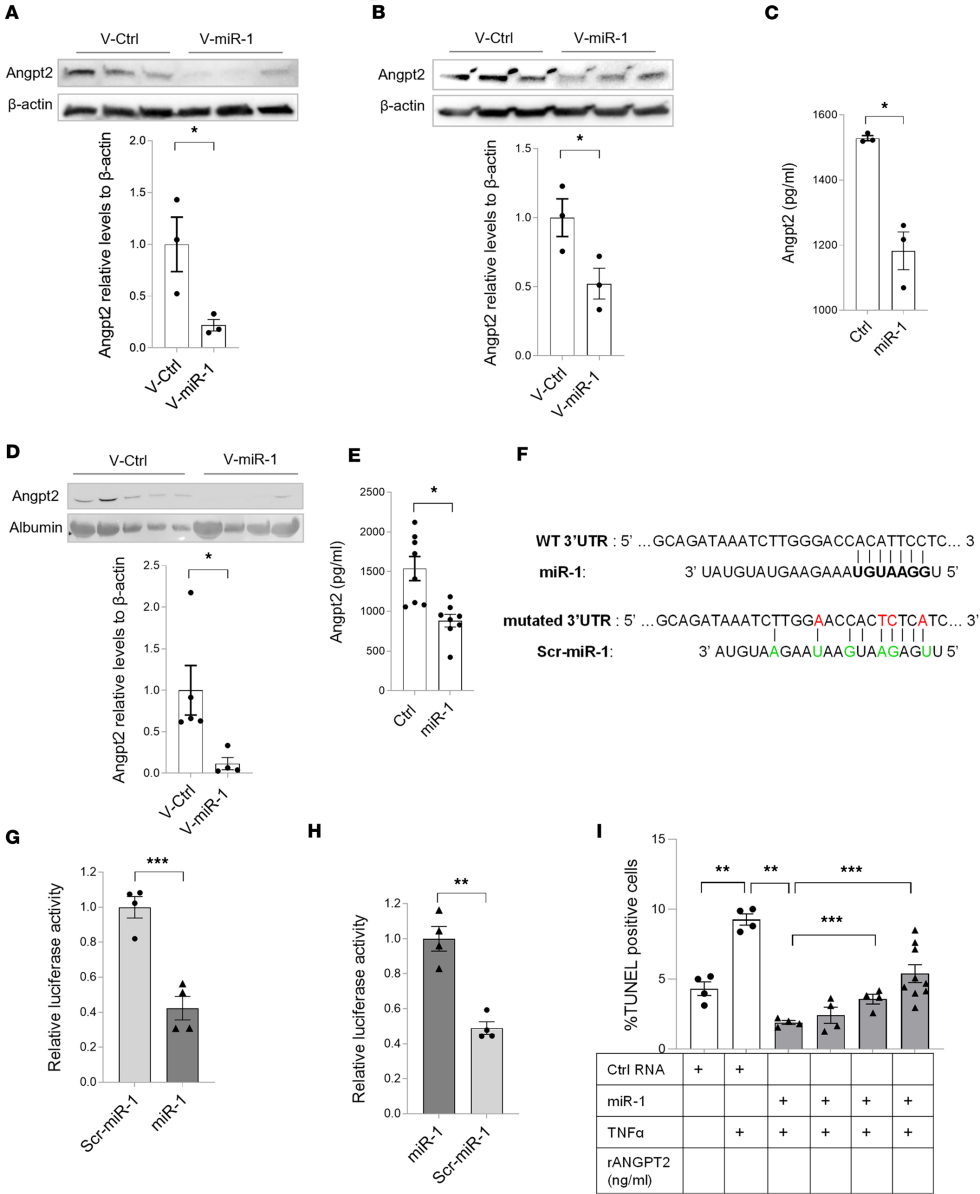
miR-1/ANGPT2 axis. (**A** and **B**) Endothelial cells were transduced with V-miR-1 or V-ctrl. Cell lysates were collected after 48 hours and ANGPT2 protein levels were measured by Western blotting. The top panels show a representative Western blot and the bottom panels show quantification based on band density values, normalized to β-actin for (**A**) HUVECs (*n* = 3, *P = 0.044) and (**B**) MLECs (*n* = 3, *P = 0.05). (**C**) HUVECs were transfected with miR-1 or control RNA (Ctrl) and grown in starvation media (containing 2% FBS) for 16 hours. ANGPT2 levels in the media were measured by ELISA (*n* = 3 per group). *P = 0.0252. (**D**) Mice received V-miR-1 or V-ctrl intranasally. BAL was collected after 2 weeks and analyzed for ANGPT2 and albumin content by Western blotting assay. The top panel shows the Western blot and the bottom panel shows band density values normalized to albumin (*n* = 5 for V-ctrl and 4 for V-miR-1). *P = 0.037. (**E**) Human lung tissue samples were cultured and transfected with a double-stranded miR-1 RNA mimic (miR-1) or control RNA (ctrl). Culture media were collected after 24 hours and ANGPT2 levels were measured by ELISA (*n* = 4 patients, 2 replicates each). *P = 0.0011. (**F**) The sequence of the miR-1 binding sites in *Angpt2* luciferase vectors: Green letters indicate mutations in the scrambled (Scr)–miR-1, and red letters indicate compensatory mutations in the *Angpt2* 3′UTR. (**G** and **H**) 293T cells were cotransfected with miR-1 or scr-miR-1 RNA and the luciferase vector containing (**G**) *Angpt2* WT 3′UTR or (**H**) *Angpt2* mutated 3′UTR. Relative firefly/Renilla luciferase activities were normalized to the mean of the control groups (2 experiments, *n* = 4). **P = 0.002, ***P = 0.0007. (**I**) HPMECs were transfected with miR-1 (or control RNA) and treated with TNF-α (10 ng/mL) in the presence of increasing concentrations of recombinant ANGPT2 and harvested after 24 hours. Cell death was measured and expressed as percentage TUNEL-positive cells, as described in Figure 4A (*n* > 4, from 2 experiments). *P = 0.01, **P < 0.0004, ***P < 0.0005). Error bars represent the SEM. Data were analyzed by unpaired, 2-tailed *t* test with Welch’s correction or Mann-Whitney *U* test based on normality.

ANGPT2, one of the proposed miR-1 targets, is a well-known mediator of endothelial dysfunction and cell death in ALI (27–29). Thus, we chose ANGPT2 for further validation studies. We first measured the levels of the translated ANGPT2 in transfected murine and human endothelial cells and their culture media. ECs in culture spontaneously synthesize and secrete ANGPT2. As shown in Figure 9, A–C, miR-1 transfection (compared with the control) significantly decreased ANGPT2 levels in the cells and in the media. Similarly, in the murine and human lungs, miR-1 overexpression (compared with the controls) decreased the secreted ANGPT2 (Figure 9, D and E). To confirm the mechanism of this downregulation, i.e., through the binding of miR-1 to its predicted binding site in the 3′UTR, we performed a luciferase assay. We cloned a 100-bp *Angpt2* 3′UTR fragment, containing the predicted miR-1 binding site, downstream of a firefly luciferase gene in pEZX-MT06, a double-luciferase expression vector, that also contains Renilla luciferase as a control gene (Figure 9F). We then compared the expression of the firefly gene relative to the control Renilla gene after cotransfection with miR-1, or its scrambled control, scr-miR-1. miR-1 reduced the relative expression of firefly luciferase by approximately 60% (compared with scr-miR-1), confirming the mechanism of miR-1–mediated inhibition and its binding site (Figure 9G). We next did a genetic compensation study to further confirm this targeting site. We introduced compensatory mutations in the *Angpt2* miR-1 binding site to make it complementary to scr-miR-1 (instead of miR-1). Using this mutated vector (mut-Angpt2) we observed that miR-1 lost its inhibitory effect and instead, cotransfection with scr-miR-1 caused 60% inhibition (Figure 9H). This result further confirmed the site and mechanism of miR-1–mediated inhibition.

We next tested the functionality of *ANGPT2* as a target of miR-1 in a cell death experiment. Since miR-1 blocks TNF-α–induced cell death (Figure 7A), we specifically asked whether supplementing ANGPT2 would reinstate the susceptibility of miR-1–transfected ECs to TNF-α. We added ANGPT2 to the media of miR-1–transfected HPMECs after TNF-α treatment and measured cell death by TUNEL assay. As shown in Figure 9I, in the presence of ANGPT2, miR-1–mediated protection was partially lost and cell death increased in a dose-dependent manner. We found similar results with HUVECs (Supplemental Figure 9). These experiments show that ANGPT2 suppression is indeed contributing to miR-1–mediated protection. They also suggest that ANGPT2 is only one of the miR-1 targets responsible for its protective effect, since even very high doses of ANGPT2 could only partially counteract the miR-1 protective effect.

### The clinical relevance of miR-1.

**Figure 10:**
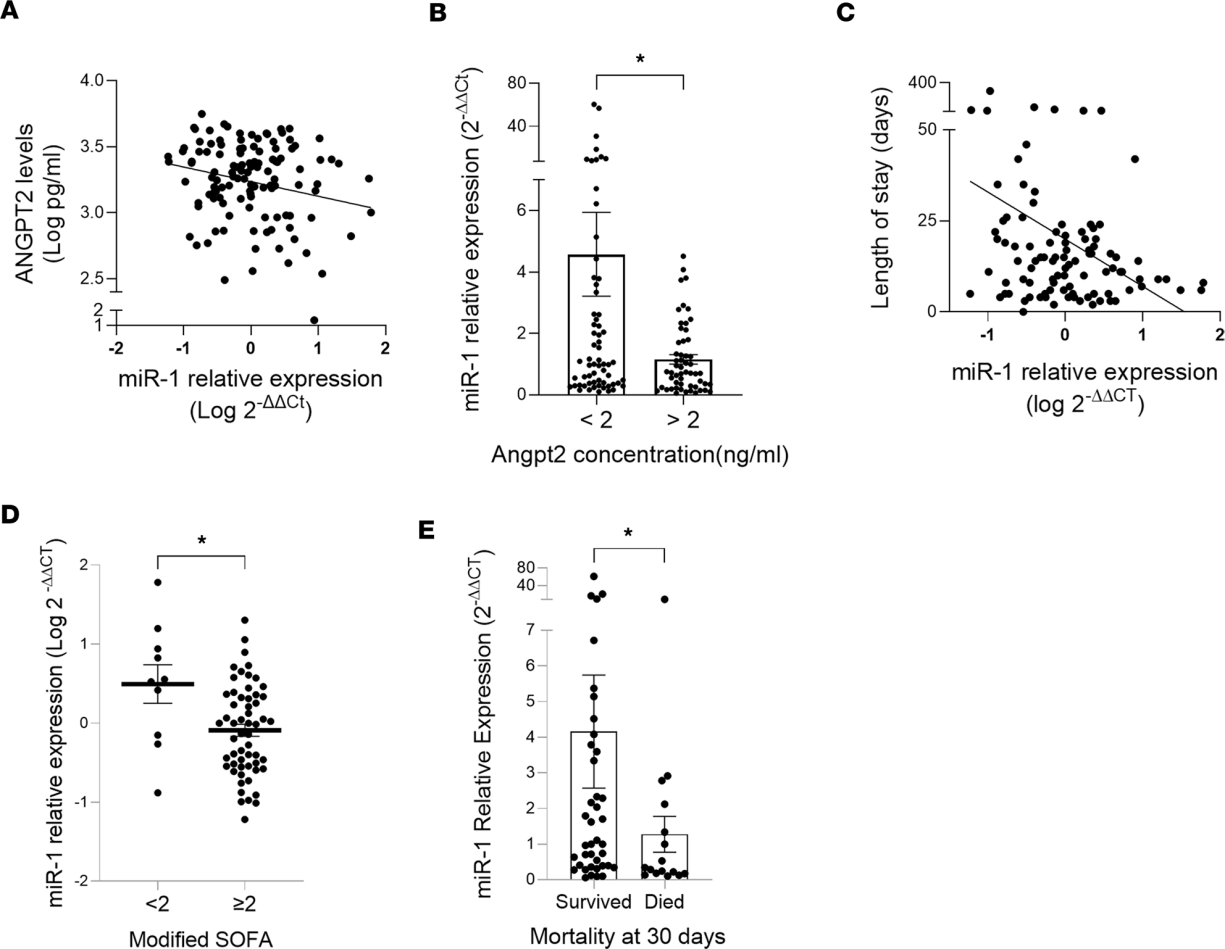
Clinical associations of miR-1 in pneumonia patients. (**A** and **B**) Serum samples were collected from hospitalized patients with bacterial pneumonia. ANGPT2 protein levels were measured by ELISA and serum miR-1/18S RNA levels were measured and expressed as in Figure 1 after normalizing to the median. (**A**) miR-1–ANGPT2 association in the whole clinical cohort (*n* = 119). Pearson’s *r* = –0.2088, *P* = 0.0227. (**B**) miR-1 levels in high-ANGPT2 (>2 ng/mL) and low-ANGPT2 (<2 ng/mL) patients (*n* = 119: 64 high- and 55 low-ANGPT2 patients). *P = 0.0462 by Mann-Whitney *U* test. (**C**) Association between miR-1 levels and the length of stay in the hospital in days (*n* = 99). Spearman’s *r* = –0.2114, *P* = 0.0357. (**D** and **E**) miR-1 levels in the patients admitted to the intensive care unit (**D**) with high (>2) or low (<2) modified sequential organ failure assessment (mSOFA) scores (*n* = 67, *P = 0.0433 by unpaired, 2-tailed *t* test with Welch’s correction) and (**E**) who had died or survived at 30 days after admission (*n* = 56, *P = 0.0450 by Mann-Whitney *U* test). Patients with do not intubate (DNI) and do not resuscitate (DNR) orders were excluded from this analysis. Error bars represent the SEM.

Since ANGPT2, a miR-1 target, is a biomarker of lung dysfunction in patients with septic shock and ARDS (27, 30–33), we performed an exploratory study to assess the clinical relevance of miR-1. We measured mature miR-1 and ANGPT2 levels in the serum samples from 119 patients who were admitted with bacterial pneumonia (Supplemental Table 3). We first assessed the correlation between miR-1 and ANGPT2 and found that, consistent with our proposed regulation, ANGPT2 had a significant inverse correlation with miR-1 (Pearson’s *r* = –0.2088, *P* = 0.0227) (Figure 10A). We then divided our cohort into high- and low-ANGPT2 groups based on the median of our ANGPT2 measurements (2.018 ng/mL) and found that high-ANGPT2 patients (compared with the low-ANGPT2 group) had significantly lower miR-1 levels in their serum (*P* = 0.0462) (Figure 10B), further confirming the inverse correlation of ANGPT2 with miR-1. Next, we assessed the correlation between miR-1 and selected indicators of disease severity in our cohort and found that miR-1 also has an inverse correlation with the length of stay (Figure 10C and Supplemental Table 4).

Most of the clinical associations for ANGPT2 have been described in critical patients (shock or respiratory failure). Therefore, we next focused on the subgroup of patients who had been admitted to the intensive care unit (Supplemental Table 5, *n* = 67). In this group of patients, apart from a stronger negative correlation with ANGPT2 (*r* = –0.3058, *P* = 0.0118), miR-1 was also inversely correlated with modified sequential organ failure assessment (mSOFA) (*r* = –0.2754, *P* = 0.0241), 30-day mortality (*r* = –0.2690, *P* = 0.0450), and length of stay (*r* = –0.326, *P* = 0.0142) (Table 1). Moreover, miR-1 levels were significantly lower in patients with an mSOFA score of greater than 2 (more severe) than those with a score of less than 2 (Figure 10D), and in survivors versus nonsurvivors (Figure 10E).

## Discussion

The main finding of this study is that during ALI, VEGF downregulates miR-1 in the lung ECs, and this downregulation increases the susceptibility of the lung to the effects of the injury. We have illustrated the specific consequences of miR-1 regulation in ALI. Isolated overexpression of miR-1 in the endothelium was adequate for lung protection in human and murine studies and increased the survival of mice after injury. miR-1 directly targeted injury-related genes in RISC and had significant correlations with ANGPT2 in ALI models, as well as in a clinical cohort.

Several miRNAs are altered in the process of ALI (7, 34). However, miRNA-mediated targeting is cell and context specific. Among the miRNAs altered in ARDS, only a few directly affect endothelium and they may enhance or ameliorate injury. miR-27a targets VE-cadherin and induces vascular leak during ischemia/reperfusion (35). miR-150 ameliorates LPS-induced permeability by targeting early growth response 2 (*EGR2*) and suppressing its downstream gene, *ANGPT2* (9), and miR-34a enhances lung endothelial injury by regulating its targets, *SIRT1* and *p53* (12). miR-1 is one of the most well-studied miRNAs in cancer and cardiac disorders (14, 32, 36). However, the specific role of miR-1 in vascular injury has never been investigated to our knowledge.

Endothelial dysfunction triggers the initiation and progression of a wide range of vascular diseases (37) and is one of the earliest events in ALI (1, 3, 38). Several vascular mediators, including VEGF, angiopoietins, endothelin-1 (39), nitric oxide (40, 41), and angiotensins (42, 43) are involved in ALI, and their levels and activities correlate with the severity of ARDS. VEGF is a potent permeability factor in the lung and increased VEGF levels predict poor outcomes after ARDS (44, 45). Also, overexpression of VEGF in the lung increases pulmonary edema and the severity of ALI (46). This seems paradoxical, as VEGF normally promotes EC survival through activation of a PI3-kinase/Akt pathway, so inhibiting VEGF expression promotes cell death (47–49). The mechanism of VEGF-mediated EC injury is therefore not clear. Our new data explain this paradox. Specifically, we found that VEGF signaling through VEGFR2 downregulates miR-1 in the injured endothelium, decreasing the capacity of ECs to withstand injury induced by other mediators such as ANGPT-2. Reports on VEGF levels during ARDS have yielded contradictory results. For instance, plasma VEGF levels increase during ARDS, while VEGF levels in the BAL, and macrophages and neutrophils isolated from the BAL of ARDS patients, were lower than controls (45, 50). However, several studies on ALI models have shown a time-wise increase in lung VEGF expression starting from the early stages of ALI (51–55). Furthermore, inhibition of VEGF expression and signaling ameliorates the extent of injury in several models (46, 56–58). A recent mechanistic study separating the endothelial and nonendothelial fractions of the injured lung concluded that during ALI, VEGF expression in ECs increases, while its expression in non-ECs and the whole lung decreases (17). The known stimulatory effect of LPS on endothelial VEGF is in line with this observation (18). Our experiments confirm the stimulatory effect of injury on endothelial VEGF and demonstrate a pathway through which VEGF, a prosurvival cytokine, can promote endothelial dysfunction in the context of ALI.

Our signaling experiments showed that PI3 kinase and JNK pathways are the main mediators of VEGF-induced miR-1 downregulation in injured ECs. The activation of the PI3 kinase pathway during various types of injury, including ALI, is well documented (59–61). Similarly, it has been shown that JNK activation is a major contributor to the injury process in hyperoxia-, paraquat-, and LPS-induced ALI models (62–64). Interestingly, however, even though p38 MAPK and ERK pathways are known to be involved in ALI (59, 65–67), the blockade of these mediators had only a partial effect on miR-1. These findings are consistent with our previous report on the miR-1 upstream pathways in angiogenesis (14) and suggest that VEGF regulates miR-1 through a distinct signaling mechanism.

We also found that injury downregulates mature miR-1 without decreasing its precursors. miRNAs are transcribed by RNA polymerase II as long primary miRNA (pri-miRNAs) transcripts that will then be cropped by Drosha complex in the nucleus to produce 60-nucelotide pre-miRNAs and exported to the cytoplasm by the transport factor exportin 5. Pre-miRNAs in the cytoplasm are cleaved and processed by Dicer into the mature 22-nucleotide miRNA duplex. One strand of this duplex (guide strand) is preferentially loaded onto one of the Argonaute (Ago1–4) proteins in RNA-induced silencing complex (miRISC) and guides it to complementary mRNAs (68). Most of the currently known miRNA regulatory mechanisms affect transcription, Drosha-mediated processing, or trimming of pre-miR-1 by Dicer (69). Our results suggest that the process of injury is not directly regulating any of these steps. Several recently discovered regulatory mechanisms affect the fate of mature miRNAs after cleavage from pre-miRNAs. Loading of miRNAs on RISC is an active ATP-dependent process and is regulated by molecular chaperones (70). Expression and stability of Ago proteins, the major components of miRISC, are also regulated by a variety of signaling pathways (71–73). Furthermore, mature miRNAs can be degraded after binding to their targets in RISC (74). Our current findings do not distinguish between these possibilities, and identifying the basic mechanism of miR-1 regulation during injury needs further investigation.

Our findings showed that miR-1 downregulation in response to injury is not limited to pulmonary infection models and occurs in other contexts and ECs. This aspect of miR-1 regulation is similar to the described activation of PI3 kinase in various other settings (61). We used the ubiquity of miR-1 downregulation during acute injury and the molecular features of miRNA-mediated targeting to identify the specific targets of miR-1 in the injured endothelium. We first chose potential targets based on their differential expression in 3 different EC types, and then selected candidate genes according to the presence of miR-1 binding sites in their 3′UTR, and finally identified the actual targets in a RISC recruitment assay. Several of these targets (*PDE5A*, *CNKSR3*, *ANGPT2*, *CX3CL1*, *IRF1*, *IRAK2*, *TNFAIP2*, and *CCL2*) are known to be involved in ALI. ANGPT2 is ubiquitously elevated in ARDS models and patients and several studies have supported the clinical value of ANGPT2 as a predictor of ARDS (7, 27, 31–33). TNFAIP2 is induced during ALI and higher levels of TNFAIP2 in patients with septic shock is associated with decreased survival (24). PDE5, CX3CL1, and CCL2 are also activated in ALI and contribute to lung dysfunction during this process (22, 23, 26, 75, 76). Targeting of these genes by miR-1 confirms its protective role and suggests that miR-1 acts as an orchestrator of the endothelial injury response during ARDS.

miR-1 exerts a cell-intrinsic effect on the endothelium, i.e., it modulates signaling pathways within the ECs that directly control their integrity. We used TNF-α to model acute endothelial injury. TNF-α is a ubiquitous mediator of ALI and has been previously used to model acute injury in pulmonary ECs (16, 77–79). The levels of TNF-α are elevated in the serum and BAL of ARDS patients (16, 80, 81), and activation of TNFR1 leads to both barrier dysfunction and cell death.

miR-1 decreased the permeability of the EC monolayers within 6 hours after TNF-α exposure, the typical time frame for increased permeability, and before the induction of significant cell death (16, 20, 82, 83). Also, miR-1 preserved the configuration of VE-cadherin and ZO-1 proteins in ECs. VE-cadherin, the main component of adherens junctions, maintains the endothelial barrier by forming intercellular homophilic interactions, and ZO-1 links VE-cadherin to tight junction proteins (84). Preservation of VE-cadherin and ZO-1 configuration by miR-1 strongly supports its protective effect against pathologic leak and the effects of miR-1 targets on the EC barrier explain this protective effect. ANGPT2 is expressed and released from activated/injured ECs (85–87) and degrades VE-cadherin by regulating its phosphorylation (28, 29, 88). Inhibition of ANGPT2 in mouse brains increases the expression of VE-cadherin and ZO-1 in the endothelium (89). Also, both *CCL2* and *LAMC2*, two other proposed targets, disrupt VE-cadherin localization in the adherens junctions (90–93).

In the later stages of ALI, ECs progress from dysfunction (leakiness) to cell death. Multiple cell death pathways are activated in ALI (3, 94–96). miR-1 inhibited caspase 3 cleavage as well as the phosphorylation of RIPK1 and MLKL, suggesting that it inhibits the apoptosis and necroptosis pathways. The inhibitory effect of miR-1 on ANGPT2 is in line with these protective effects. ANGPT2 sensitizes ECs to TNF-α and in the setting of lung infection/injury, promotes cell death through TIE-2–dependent and –independent pathways (27, 87, 97). *Angpt2* expression in the lung is necessary for caspase 3 activation after hypoxic injury (27), and a recent study on ARDS patients showed an association between ANGPT2 and the activation of MLKL (98). Our supplementation/rescue experiment (Figure 9I) confirmed the role of ANGPT2 in miR-1 protection against cell death. It also suggested that ANGPT2 is not the only mediator of this protection and other targets are also contributing. In accord with this finding, other miR-1 targets are known to regulate cell death pathways. *TNFAIP2* is induced in the ECs by LPS and a variety of inflammatory cytokines (99), promotes apoptosis (100, 101), and can activate caspase 3/7 (102). *PDE5A*, which encodes the most well-studied guanosine 3′,5′-cyclic monophosphate (cGMP) esterase, is expressed in the endothelium and has a proapoptotic effect on pulmonary ECs (103). Interestingly, *PDE5* inhibition protects mitochondrial function (104), further supporting its potential role in acute endothelial injury. CNKSR3 is also proapoptotic and regulates caspase 3 cleavage (25, 105).

The well-known value of ANGPT2 as a predictor of ARDS severity (33, 75, 106, 107) strongly suggested that miR-1 may have relevance in clinical settings. We thus validated ANGPT2 targeting in our human models and tested the correlations of miR-1 in our pneumonia cohort. Interestingly, apart from ANGPT2, miR-1 also showed statistically significant associations with several indices of ARDS severity, such as 30-day mortality. Although the scope of our clinical study is limited, these exploratory analyses support the likely role of miR-1 in the pathogenesis of ARDS.

Our findings indicate that miR-1 primarily protects the endothelium. However, these observations do not rule out the possibility of protective effects on other cell types. Changes in the endothelium can alter immune cell trafficking and both *CCL2* and *CX3CL1*, targets of miR-1, affect leukocyte transmigration (108, 109). Also, endothelial miR-1 may protect the neighboring epithelium through a paracrine mechanism. Cross-signaling between the lung endothelium and epithelium occurs in various contexts, including ALI (1, 110), and angiocrine molecules, such as ANGPT2, are known to mediate such interactions. Furthermore, accumulation of edema fluid and circulatory failure caused by endothelial dysfunction can lead to secondary injury and death in the adjacent cells.

Endothelial dysfunction is a central pathophysiological event in ARDS (1–3). Regarding the seminal role of the vasculature in orchestrating the response to injury, decoding the molecular underpinnings of endothelial dysfunction is a necessity. In this study, we found that endothelial miR-1 levels decrease in acute injury and that increasing miR-1 levels protects the endothelium and the whole lung. Furthermore, miR-1 downregulation in other ECs and types of injury suggests a universal role for miR-1 in the injured endothelium. Our exploratory study on patients with pneumonia suggests that our findings may have clinical relevance. We thus propose the miR-1 axis as an injury-related gene network with pathogenic, prognostic, and therapeutic potential.

## Methods

### Cell culture.

Primary ECs were used up to passage 7 (passage 4 for HUVECs). Details on EC growth and cell-based injury models are available in the supplemental material.

### Murine models.

A description of the murine injury models is available in the supplemental material.

### Measurement of lung water.

Extravascular lung water was measured as previously described (111). Details are available in the supplemental material.

### Measurement of endothelial permeability.

Endothelial permeability was measured using ECIS from Applied BioPhysics. A detailed description is available in the supplemental material.

### Measurement of ANGPT2.

ANGPT2 levels in culture media or patients’ sera were measured by ELISA (Human Duoset, R&D Systems). One hundred microliters of serum or cell media and 3-hour incubation at room temperature were used for initial antigen capture.

### PCLS.

Human PCLSs were generated as previously described (112–114). Details are available in the supplemental material.

### Comparative analysis for miR-1 targets in ALI.

HPMECs, HUVECs, and HDMECs were treated with TNF-α (1 ng/mL; 210-TA-020 CF, R&D Systems) for 6 hours. Total RNA was extracted using TRIzol (16096040, Thermo Fisher Scientific) and the miRNeasy Kit (Qiagen). mRNA levels were analyzed by RNA sequencing (115). Genes with differential expression above 0.5 log(fold change) and *P* value less than 0.05 were considered for further analysis. miR-1 targets were identified using miRcode (http://www.mircode.org/), miRTarBase (https://mirtarbase.cuhk.edu.cn/~miRTarBase/miRTarBase_2022/php/index.php), miRDB (http://www.mirdb.org/), and miRWalk (http://mirwalk.umm.uni-heidelberg.de/) algorithms.

### RISC recruitment (Argonaute pull-down) assay.

HUVECs were transduced with V-miR-1 (or control, V-ctrl) and subjected to Argonaute-2 RNA immunoprecipitation assay followed by small sequencing, as described previously (15). Validation of mRNA levels in input and immunoprecipitate samples was performed using SYBR Green qRT-PCR (Bio-Rad).

### Luciferase assay.

*Angpt2* luciferase vectors were cloned using the pEZX-MT06 vector (GeneCopoeia, a gift from Maor Sauler, Yale University, New Haven, Connecticut, USA) and luciferase assays were performed using Luc-Pair Duo-Luciferase HS Assay Kit (LF004, GeneCopoeia). A detailed description of the vectors and the luciferase assays are available in the supplemental material.

### Clinical pneumonia cohort.

Serum samples were obtained from 119 patients who were admitted to Yale New Haven Hospital (IRB 0901004619) between 2004 and 2007 and diagnosed with pneumonia based on a positive respiratory culture. A detailed description of the cohort is available in the supplemental material.

### Statistics.

Statistical analyses were performed using GraphPad Prism software version 9.4.0. The normality (Gaussian distribution) of the data sets was tested using the D’Agostino-Pearson or Kolmogorov-Smirnov test. Parametric tests were used for normally distributed data. Normally distributed continuous variables were compared using the Student’s *t* test with Welch’s correction. Skewed-distributed continuous variables were compared using the Mann-Whitney *U* test. Clinical correlations were analyzed by Pearson’s test for parametric data and Spearman’s for nonparametric data. Kaplan-Meier curves for the survival of mice were analyzed using the log-rank (Mantel-Cox) test. Line bars represent the mean of the group and error bars represent the standard error of mean (SEM).

### Study approval.

All human tissue and serum samples were used after receiving written informed consent from patients and after the approval by Yale Human Investigation Committee and Institutional Review Board (HIC protocol 1103008160 and IRB 0901004619). The animal protocol was approved by Institutional Animal Care & Use Committee (IACUC) at Yale University (protocol 2021-20050).

### Data availability.

RNA-sequencing data were deposited in the NCBI Gene Expression Omnibus (GEO) database under accession numbers GSE190181, GSE161021 (115), GSE236263, and GSE239928. Values for all data points found in graphs can be found in the Supporting Data Values file and are available from the corresponding author upon request.

## Author contributions

A Korde, MH, A Khan, and SST performed experimental work. A Korde and PP collected clinical data. A Korde, PP, and SST analyzed clinical data. SST and A Korde wrote, designed, and edited the manuscript. SM and FLG analyzed RNA-sequencing data. A Korde and MC performed microscopy. RWP performed experimental work and provided reagents. MR and CDC provided clinical samples. SB collected and processed pneumonia samples. JSP and MAM reviewed and edited the manuscript.

## Supplementary Material

Supplemental data

Supporting data values

## Figures and Tables

**Table 1:**
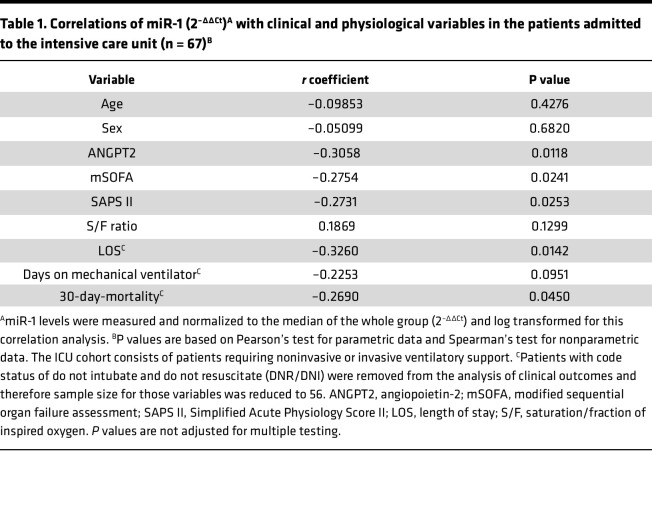
Correlations of miR-1 (2^–ΔΔCt^)^A^ with clinical and physiological variables in the patients admitted to the intensive care unit (n = 67)^B^

## Version 1 09/22/2023

Electronic publication
