# Supplementary material for: MicroRNA-1 protects the endothelium in acute lung injury
Source: JCI Insight. 2023 Sep 22;8(18):e164816. doi: 10.1172/jci.insight.164816 (PMC10561733; doi:10.1172/jci.insight.164816)
Supplement: Supplemental data [file jciinsight-8-164816-s222.pdf]

Supplemental methods

Cell culture and Cell-based injury models: HPMECs and HDMECs were isolated by immunoselection using CD31 magnetic microbeads (Miltenyi) (1) and grown in Endothelial Cell Growth Medium-2 with Bullet Kit (Lonza). HUVECs were purchased from Vascular Biology Therapeutics, Yale University, and cultured in the M199 (Gibco) medium containing 20% FBS (Heat inactivated, Gibco), 1% ECGS, and 1% penicillin, and streptomycin. Primary endothelial cells were used up to passage 7 (passage 4 for HUVEC). HEK293T (ATCC, CRL-11268) cells were cultured in Dulbecco's Modified Eagle's Medium (DMEM) with 10% FBS and 1% penicillin, and streptomycin.

For cell death and injury, HUVECs and HPMECs were serum starved for 8 hours in minimal media M199 or EBM2 media (Lonza cat # CC-3156) and then treated with TNFα or LPS for 24 hours in their growth media. Endothelial cells and ex vivo tissues were transfected with miR-1 double stranded mimic or control RNA at a concentration of 50-100 ηM per reaction using Continuum™ Transfection Reagent (Gemini Bio-products, cat # 400-700) for 24-36 hours. MiR-1 sense and antisense RNA oligos were synthesized (Integrated DNA technologies, IDT) and hybridized (95 C for 1’ and 37 C for 30’) in 1x siRNA buffer and diluted with RNase-free water to obtain 50-100 μM concentration. Cells and tissues were transduced with V-miR-1 or V-ctrl lentiviral vectors (MOI=0.8). After 36 hours transduction efficiency was confirmed by checking the fluorescence [from the GFP cassette in the vector, as described before, (2)] in the cells. In blocker experiments, HPMECs were seeded, starved in EBM2 basal media with 2%FBS for 6-8 hours and treated with blockers for 2 hrs before TNFα treatment. Blockers: U0126 (MEK1 and 2 inhibitor, Promega, used as ERK inhibitor and solubilized in DMSO) at 15uM final concentration, SB203580 (P38 MAP Kinase inhibitor, solubilized in DMSO) at 10uM final concentration, SP600125 (JNK inhibitor, Sigma, solubilized in water) at 20 μM final concentration, Ly294002 (PI3 kinase inhibitor, Millipore,

solubilized in DMSO) at 50uM final concentration were added to the cells 1-2 hours before adding TNFα at 10 ng/ml. Cells were lysed 20-24 hours after the addition of TNF-α.

Murine injury models: In all models, 6–8-week-old C57BL/6J male mice (Jackson Laboratory) received intranasal V-miR-1 (or V-ctrl control) lentivirus. For miR-1 transgenics, transgene was induced by adding Doxycycline (1 g/L) to the drinking water. Two weeks after lentiviral delivery or 4-5 days after inducing miR-1 transgene, the mice were exposed to the injurious agent. *E.coli model*: mice were infected intranasally with 30-40 μL of bacterial suspension containing 10^9-10^{10} colony-forming units of E. coli in PBS. and lungs and BAL were collected after 24 hours. *LPS model:* Mice were given intranasal LPS (4mg/kg), and lungs and BAL were collected after 24 hours. *Hyperoxia:* Mice were kept in a hyperoxia chamber containing 95% oxygen. Lungs and BAL were collected after 72 hours.

Ex-vivo cultured human lung injury model: Histologically normal lung tissue samples were obtained from patients who underwent surgical resection for lung masses at the Yale Cancer Center. All tissue samples were used after approval from the Yale Human Investigation Committee that approved the design of the study and patient consent forms (HIC protocol # 1103008160). Patients were consented for gene expression measurements before undergoing the surgical procedure. Fresh samples were kept in DMEM (Dulbecco Minimal Essential Medium) supplemented with 10% fetal Bovine Serum (FBS) at 4 degrees for up to 12 hours before being delivered to the lab for ex vivo culture. The lung tissue was cut into approximately 3-4mm size pieces and cultured in M199 (Life Technologies), 20% FBS (Gibco, Life Technologies) at 37 C. Tissues were treated with 500 ng/ml of LPS (E.coli, Sigma) or vehicle (phosphate buffered saline, PBS). Tissue samples and media were harvested after 24 hours. For RNA expression analysis tissue samples were stored in Trizol at -80 C and total RNA were isolated using Trizol followed by phenol/chloroform extraction, per the manufacturer's protocol. The concentration and quality of these RNA samples were determined using Nanodrop spectrophotometer (Thermo Scientific).

Measurement of miR-1: Mature miR-1 levels were measured by quantitative stem loop RT-PCR either using Taqman (life Technologies) or SYBR green (BioRad) reagents. Reverse transcription was performed using the miR-1 RT primers and miR-1 specific primers forward and reverse primers were used for quantitative real time PCR as described before (2, 3). We used comparative Ct (cycle threshold) method to analyze miR-1 expression in samples. The relative expression of a gene (miR-1 or other mRNA) in a sample is first calculated by dividing the 2^{Ct} value of the target gene by the 2^{Ct} value of an internal control (RNU48 or 18S) to obtain $2^{-\Delta Ct}$ value (4). In some instances, for the simplified version values are expressed as $2^{-\Delta\Delta Ct}$ after dividing the $2^{-\Delta Ct}$ value for a target gene by the mean of $2^{-\Delta Ct}$ values for the experimental control samples (PBS, '0' concentration group or healthy subjects).

Measurement of lung water: Right ventricular puncture was performed on mice to collect blood in EDTA tube. 150ul of blood was weighed. Right lungs were weighed and homogenized in 1 ml double distilled water. 200ul of homogenate was weighed separately and remaining homogenate was centrifuged at 14,000 rpm for 10 mins. The pellet was discarded and 200ul of supernatant was weighed. Aliquots of blood, lung homogenate and supernatant were placed in drying oven at ~60 C for 24 hours and then weighed again.

Precision Cut Lung Slices (PCLS): The human lung segments (obtained through the National Disease Research Interchange [NDRI]) was inflated with warm buffer-equilibrated low melting agarose (3%) via the bronchus. Segments were cooled at 4°C for 30 minutes and cryopreserved in liquid nitrogen. Later, PCLS were prepared to a thickness of 300 μm using a Compresstome (VF-300-0Z by Precisionary®) at cutting speed of 6 μm s^{-1} and oscillation frequency of 5Hz and cultured in M199 medium containing 20% FBS, 1% ECGS and 1% penicillin and streptomycin in 12-well plates at 37 °C, 5% CO2 and 95–100% air humidity under tissue culture conditions. PCLS were transduced with V-miR-1 or control (V-ctrl) and exposed to 500ng/ml LPS, 36 hours after transduction. After 24 hours of LPS exposure samples were fixed in 10% NBF and 70% ethanol

and embedded in paraffine. 5-7 μm sections were placed on slides and immunostained with anti-VWF (1:500, for endothelial cells, Cat# A0082, Dako), anti-EPCAM (1:300, for epithelial cells, Cat #17-5791-82, Invitrogen), followed by TUNEL staining (Roche Applied Science) according to the manufacturer's protocol. Images were taken either on 2-photon microscope or confocal microscope (Leica SP5) at the Imaging core of Yale School of Medicine. TUNEL positive cells were counted per alveolar length or vessel perimeter.

Immunostaining for adherence and tight junction proteins: HPMEC were grown to confluency and monolayer was established on gelatin-coated coverslips in 24-well cell culture plate. In the plate cells were washed with PBS containing 100mg/L of calcium and magnesium, then fixed in 95% ethanol for 30 min at 4°C. Cells were immunostained for anti-VE-Cad (R&D systems, #AF938) or anti-ZO-1 (Santa Cruz, sc-33725) with 1:100 antibody dilution. Glass coverslips were mounted for confocal immunofluorescence analysis in ProLong mounting media with 4′,6-diamidino-2-phenylindole (DAPI, Invitrogen) and imaged using Leica SP5 confocal microscope as previously describe (1).

Measurement of cell death pathway proteins (western): HPMECs were transfected with miR-1 RNA mimic (miR-1) or control RNA (Ctrl) for 24 hours in a growth medium, followed by 24 hour exposure to TNFα (10 ng/ml). Cells were harvested for protein lysates with 100 ul Laemmli buffer containing β-mercaptoethanol. 25-35ug of protein were loaded for western blotting. Antibodies were purchased from Cell Signalig Technologies. RIP (Cat# 3493T), phospho-RIP (Cat# 65746S), MLKL (Cat# 14993S), phospho-MLKL (Cat# 91689S), CASP3 (Cat# 14220T), Cleaved CASP3 (Cat# 9664T), Gasdermin D (Cat# 93709) and cleaved Gasdermin D (Cat# 36425). All antibodies were diluted at 1:1000 concentrations except cleaved CASP3 (1:500).

Measurement of cell death (in human and murine lung and endothelial cells):

LDH: We measured lactate dehydrogenase (LDH) activity released from the cytosol of damaged cells into the cell culture media or mouse BAL using Cytotoxicity Detection Kit^{PLUS} (LDH) (4744926001, Roche Millipore Sigma).

TUNEL: We used the I*n Situ* Cell Death Detection Kit according to the manufacturer's protocol (Roche Applied Science). For lung tissues, sections of formalin-fixed, paraffin embedded tissues were deparaffinized and rehydrated, rinsed with PBS, and digested with proteinase K (Roche Applied Science) at a concentration of 20 mg/ml for 20 min. For endothelial cells, cells were fixed in 4% PFA for 15 mins at RT, followed by permeabilization on ice for 5 mins by 0.1% Triton-100 in 0.1% sodium citrate. After PBS washes, sections were incubated with TUNEL reaction mixture at 37°C for 120 mins. Sections were washed twice with PBS and a drop of Vectashield with DAPI was used as a mounting medium. Green fluorescent apoptotic (TUNEL positive) cells were observed under a florescent microscope. Total cells were counted using DAPI. The percentages of TUNEL-positive cells (green nuclei, apoptotic cells)/total cells (DAPI-positive cells, blue) were calculated and graphed. Images were collected at 200X magnification.

FACS: Cellular apoptosis was evaluated using an Annexin V/propidium iodide (PI)-staining kit (Thermo Fisher Scientific) following the manufacturer's instructions. Single cell suspension from miR-1 TG (or WT) murine lungs were stained with anti-CD45 (PerCP Cy5.5, BD Pharmingen #550994), followed by anti-CD31 (APC, Biolegend #102509) and Annexin V/PI antibodies. The percentage of dead (Annexin V and PI-positive) endothelial (CD31+, CD45-) cells were analyzed by flow cytometry and plots are made using flow jo v10.7.2 software.

Measurement of endothelial permeability- ECIS, Immunofluorescence for EC integrity: Endothelial permeability was measured by Monitoring the Barrier Function of Cell Monolayers using ECIS (Electric Cell-substrate Impedance Sensing) from Applied BioPhysics. Briefly, 8- or

96-well ECIS plate was treated with L-cysteine and then coated with 0.1% gelatin. HPMECs transfected with miR-1 RNA mimic (miR-1) or control RNA (Ctrl) for 24-36 hours in a growth medium**.** Transfected cells were seeded in pre-treated ECIS plate with a high density 40,000-60,000 cells/cm^2 to obtain confluency at least after 48 hours. Then, initial resistance was checked to confirm stable barrier. After reaching the resistance of ~1500 ohms, the experiment was started by adding TNFα (10ng/ml) or vehicle (PBS) to the media and recording was continued for up to 12 hours afterwards. Measurements over time were plotted after normalizing to the baseline values, which were set at 1.0.

Luciferase assay: *Angpt2* 100-bp 3'UTR sequence (containing miR-1 binding site) was synthesized using oligos with EcoRI and SpeI sequences at the ends, followed by hybridization to make a double-stranded DNA insert. Luciferase vector pEZX-MT06 (GeneCopoeia, a gift from Maor Sauler, Yale University, New Haven) was opened by releasing the EcoRI/SpeI fragment and the Angpt2 insert was ligated using T4 DNA ligase (New England Biolab) followed by TOPO cloning (Invitrogen). Colonies were checked by EcoRI/SpeI digestion and DNA sequencing. *Angpt2* mutated 3' UTR luciferase vector was created as described above using a synthesized double-stranded DNA insert containing the mutated sequence. 293T cells were co-transfected with the luciferase vector containing either *Angpt2* wild type (WT) or mutated (MUT) 3'UTR, and miR-1 or Scr–miR-1 RNA. Luciferase assay was performed using Luc-Pair™Duo-LuciferaseHSAssayKit (GeneCopoeia). Relative luciferase activity (firefly/renilla luciferase expression) was measured with a luminometer (BioTek Cytation 3) and expressed as relative luciferase activity after normalizing to the mean of their respective control groups.

Pneumonia cohort: Patients with age<18 years and pregnancy were excluded from the study. Total RNAs were extracted from 100ul of sera by phenol-chloroform extraction and miR-1 was measured by stem-loop PCR. ANGPT2 in serum was measured by ELISA (Cat# DY623, R&D systems). Demographic characteristics such as age, gender, smoking status, prior medical diagnosis of significance as well as biochemical indices (accurate to the day of collection of miR-

1 serum sample) and clinical outcome data such as survival, supplementary oxygen, mechanical ventilation, overall length of stay were collected by chart review. Clinical indices of severity were calculated using modified SOFA score and SAPS 2 score for all patients as previously described (5-7). A score of ≥2 was used for clinically significant organ dysfunction as previously described (8-11).

Sequencing analysis: HPMEC followed the same growth, treatment and sequencing protocol as HDMEC (PMID: 33948992) and HUVEC (PMID: 35294066)cells. Replicate cultures of HPMECs were maintained for 2-day post-confluence in a 12 well plate (Falcon) and stimulated with 0.5 ng/mL TNF (Invitrogen) for 6 hours or left untreated. Cell monolayers were washed with sterile phosphate-buffered saline (PBS, Invitrogen) and total RNA was purified using the RNeasy Mini Kit (Qiagen) with an on-column DNase treatment. Unless otherwise specified, experiments were performed in triplicate. RNA integrity analysis, library preparation, and sequencing were performed by the Yale Center for Genomic Analysis. RNA integrity was measured using an Agilent 2000 Bioanalyzer utilizing the Agilent RNA 6000 Pico Chip (Agilent, Santa Clara, CA) per the manufacturer's specifications. Library preparation was performed with the Illumina TrueSeq Library Preparation Kit (Illumina, San Diego, CA) per the manufacturer's specifications. Following first-strand synthesis with random primers, second-strand synthesis was performed with dUTP for generating strand-specific sequencing libraries. For whole genome sequencing, libraries were sequenced on an Illumina HiSeq2500 with parameters set for high output, single-end chemistry, and 76-bp sequencing as previously described (12).

Sequencing reads for each cell type were processed together. For each read, we trimmed the first 6 nucleotides and the last nucleotides at the point at which the Phred score of an examined base fell below 20 using in-house scripts. If, after trimming, the read was shorter than 45 bp, the whole read was discarded. Trimmed reads were mapped to the human reference genome (hg19) with a known transcriptome index (UCSC Known Gene annotation) with Tophat v2.1.1 (13) using

the very-sensitive preset, first-strand library type and providing the corresponding gene model annotation. In these results, only the reads that mapped to a single genomic unique location, with a maximum of two mismatches in the anchor region of the spliced alignment, were reported. The default settings for all other Tophat options were used. The program Cufflinks v2.2.1 (14) was used to convert aligned reads generated from Tophat into relative expression values for each gene represented as FPKM (fragments per kilobase of exon per million mapped reads). Cuffdiff was used to obtain differential gene expression between the experimental groups using first-strand library type, providing gene model annotation and the genome sequence file for detection and correction of sequence-specific bias that can be caused by random hexamer during the process of library preparation. Sequencing data for the HDMECs and HUVECs have been previously deposited in NCBI's Gene Expression Omnibus (GSE161021 and GSE190181, respectively). New HPMEC sequencing and Ago-2 pull-down sequencing results for this study are deposited in GEO database under accession numbers GSE236263 and GSE239928, respectively.

1. Khan A, Ni W, Baltazar T, Lopez-Giraldez F, Pober JS, and Pierce RW. ArhGEF12 activates Rap1A and not RhoA in human dermal microvascular endothelial cells to reduce tumor necrosis factor-induced leak. *The FASEB Journal.* 2022;36(4):e22254.
2. Korde A, Jin L, Zhang J-g, Ramaswamy A, Hu B, Kolahian S, Guardela BJ, Herazo-Maya J, Siegfried JM, and Stabile L. Lung endothelial microRNA-1 regulates tumor growth and angiogenesis. *American journal of respiratory and critical care medicine.* 2017;196(11):1443-55.
3. Korde A, Ahangari F, Haslip M, Zhang X, Liu Q, Cohn L, Gomez JL, Chupp G, Pober JS, and Gonzalez A. An endothelial microRNA-1–regulated network controls eosinophil trafficking in asthma and chronic rhinosinusitis. *Journal of Allergy and Clinical Immunology.* 2020;145(2):550-62.
4. Huggett J, Dheda K, Bustin S, and Zumla A. Real-time RT-PCR normalisation; strategies and considerations. *Genes & Immunity.* 2005;6(4):279-84.
5. Grissom CK, Brown SM, Kuttler KG, Boltax JP, Jones J, Jephson AR, and Orme JF. A modified sequential organ failure assessment score for critical care triage. *Disaster medicine and public health preparedness.* 2010;4(4):277-84.
6. Rahmatinejad Z, Reihani H, Tohidinezhad F, Rahmatinejad F, Peyravi S, Pourmand A, Abu-Hanna A, and Eslami S. Predictive performance of the SOFA and mSOFA scoring systems for predicting in-hospital mortality in the emergency department. *The American journal of emergency medicine.* 2019;37(7):1237-41.
7. Pandharipande PP, Shintani AK, Hagerman HE, St Jacques PJ, Rice TW, Sanders NW, Ware LB, Bernard GR, and Ely EW. Derivation and validation of SpO2/FiO2 ratio to impute for PaO2/FiO2 ratio in the respiratory component of the Sequential Organ Failure Assessment (SOFA) Score. *Critical care medicine.* 2009;37(4):1317.
8. Singer M, Deutschman CS, Seymour CW, Shankar-Hari M, Annane D, Bauer M, Bellomo R, Bernard GR, Chiche J-D, Coopersmith CM, et al. The Third International Consensus Definitions for Sepsis and Septic Shock (Sepsis-3). *JAMA.* 2016;315(8):801-10.
9. Hantrakun V, Somayaji R, Teparrukkul P, Boonsri C, Rudd K, Day NP, West TE, and Limmathurotsakul D. Clinical epidemiology and outcomes of community acquired infection and sepsis among hospitalized patients in a resource limited setting in Northeast Thailand: A prospective observational study (Ubon-sepsis). *PLoS One.* 2018;13(9):e0204509.
10. Wright SW, Lovelace-Macon L, Hantrakun V, Rudd KE, Teparrukkul P, Kosamo S, Liles WC, Limmathurotsakul D, and West TE. sTREM-1 predicts mortality in hospitalized patients with infection in a tropical, middle-income country. *BMC medicine.* 2020;18(1):1-9.
11. Gholipour Baradari A, Sharifi H, Firouzian A, Daneshiyan M, Aarabi M, Talebiyan Kiakolaye Y, Nouraei SM, Zamani Kiasari A, Habibi MR, and Emami Zeydi A. Comparison of proposed modified and original sequential organ failure assessment scores in predicting ICU mortality: A prospective, observational, follow-up study. *Scientifica.* 2016;2016(
12. Serebrenik YV, Hellerschmied D, Toure M, López-Giráldez F, Brookner D, and Crews CM. Targeted protein unfolding uncovers a Golgi-specific transcriptional stress response. *Molecular Biology of the Cell.* 2018;29(11):1284-98.
13. Trapnell C, Pachter L, and Salzberg SL. TopHat: discovering splice junctions with RNA-Seq. *Bioinformatics.* 2009;25(9):1105-11.
14. Trapnell C, Williams BA, Pertea G, Mortazavi A, Kwan G, Van Baren MJ, Salzberg SL, Wold BJ, and Pachter L. Transcript assembly and quantification by RNA-Seq reveals unannotated transcripts and isoform switching during cell differentiation. *Nature biotechnology.* 2010;28(5):511-5.

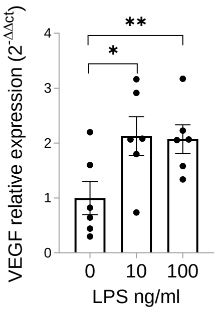

Figure S1: Endothelial cells were treated with increasing concentrations of LPS for 24 hrs and miR-1/18S levels were measured by stem-loop PCR, analyzed by comparative Ct method, normalized to the levels in ‘0’ concentration groups and expressed as $2^{-\Delta\Delta Ct}$ **(C)** VEGF levels were measured in HPMECs exposed to LPS as described in ‘Figure 2F’. (n=6, *p=0.036, **p=0.023)

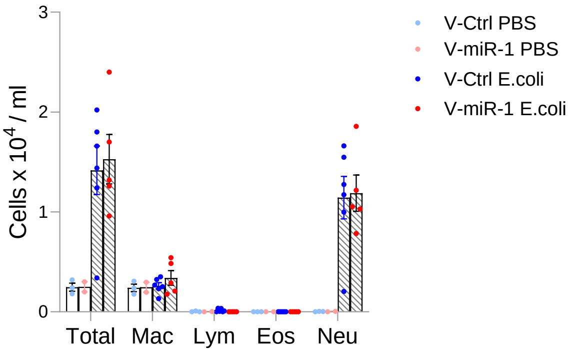

Figure S2: Mice received endothelial specific lentiviral vectors V-miR-1 or control (V-ctrl) and infected with E.coli through intranasal route. BAL was collected 24 hours post-infection. Macropgages (Mac) Lymphocytes (Lym), eosinophils (Eos), neutrophils (Neu) were counted after differential HEMA 3 staining.

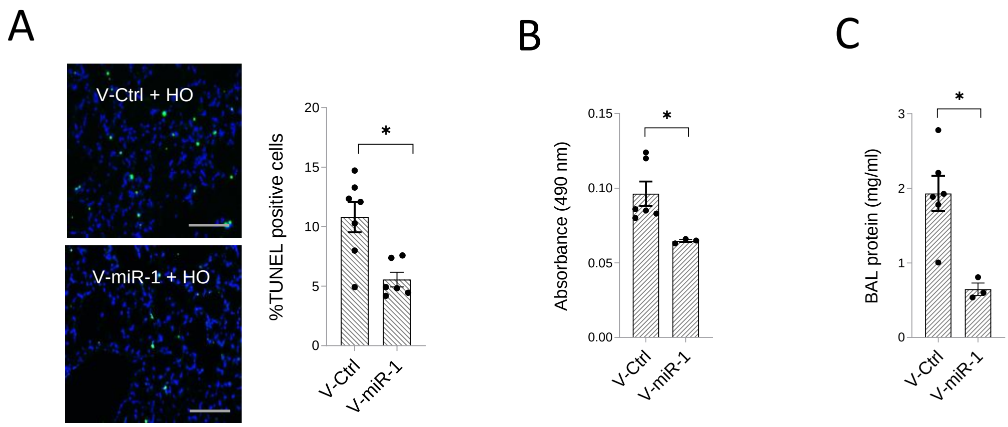

Figure S3: Mice received endothelial specific lentiviral vectors V-miR-1 or control (V-ctrl) and subjected to hyperoxia. After 72 hours BAL was collected, and lungs were harvested. **a)** TUNEL assay was performed and %TUNEL-positive cells were measured and graphed as in 3a. Data from one representative experiment. (n=6 or more per group, *p=0.005). Grey Scale bar= 100μm.) **b)** LDH was measured in BAL (n=6 for V-ctrl and n=3 for V-miR-1, *p=0.0033). **c)**Total protein was measured in the BAL. (n=6 for V-ctrl and n=3 for V-miR-1, *p=0.0032). [HO= hyperoxia]. Error bars represent standard error of mean.

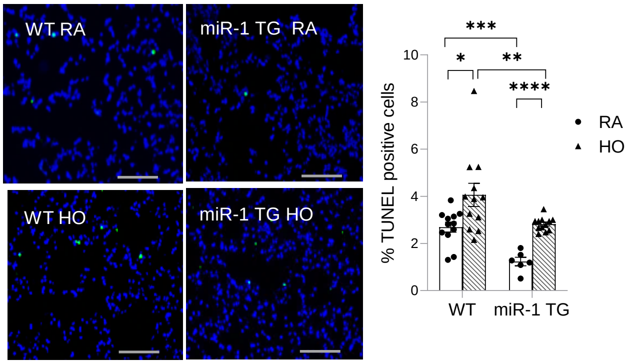

Figure S4: Hyperoxia exposure in miR-1 transgenic (miR-1 TG) and wild type (WT) mice: MiR-1 transgene was induced by adding Doxycycline (1g/L) to the drinking water for 5 days. Mice were then exposed to hyperoxia (HO) or room air (RA). Lungs were harvested after 72 hours and TUNEL-positive cells measured and graphed as in 3a (n=4,5; *p=0.0423, **p=0.05, ***p<0.0001, ****p<0.0001). Scale bar=100µm. Error bars represent standard error of mean.

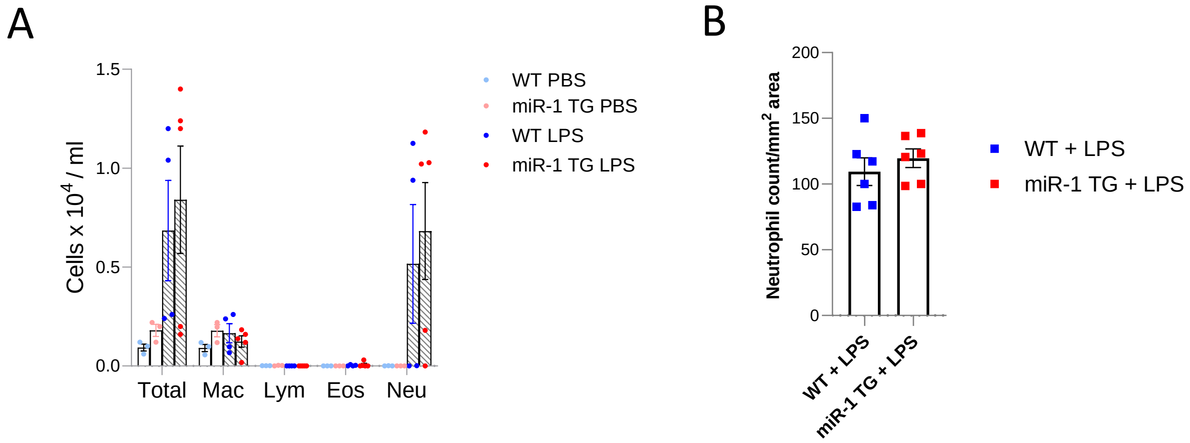

Figure S5: MiR-1 TG (or WT) mice were exposed to 4mg/kg LPS through intratranasal route 5 days after induction of miR-1 transgene. BAL and lungs were harvested 24 hours after LPS delivery. **(A)** Macropgages (Mac) Lymphocytes (Lym), eosinophils (Eos), neutrophils (Neu) were counted in BAL after differential HEMA 3 staining (representative data for 2 experiments). **(B)** Paraffin-embedded lung sections were stained with H&E and number of neutrophils were counts in peribronchial and alveolar regions. (representative data for 2 experiments, n=6, p=ns)

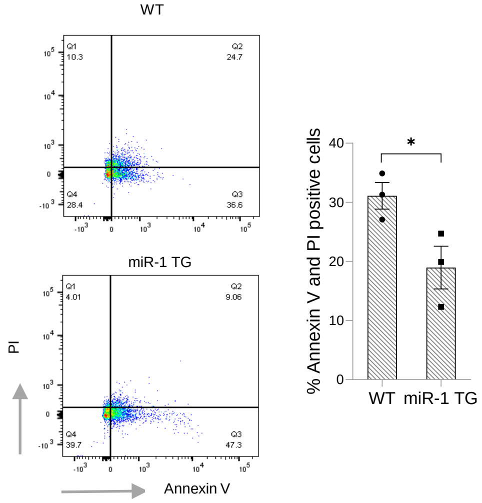

Figure S6: MiR-1 transgenic mice (miR-1 TG) and their wild type litters (WT) were treated with LPS (or vehicle, PBS) and their lungs harvested after 24 hours, as described in 3g. Single cell suspensions of these lungs were stained with anti-CD31, andti-CD45 and Annexin V/PI antibodies and the percentage of dead (Annexin V and PI-positive) endothelial (CD31+, CD45-) cells were analyzed by flow cytometry. The panel on the right shows representative scatter-plots and the graph on the left shows the values for each group. (n=3 for LPS groups, *p=0.03). Error bars represent standard error of the mean.

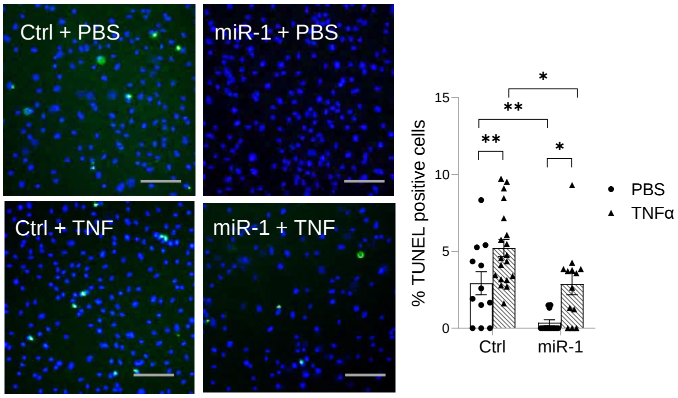

Figure S7: HUVECs transfected with miR-1 or control RNA (Ctrl) and after 24 hours, treated with TNF-α (10ng/ml) or vehicle (PBS) for another 24 hours. %TUNEL positive cells were determined as in 3a. Scale bar= 60μm (n=4, *p<0.04, **p<0.003). Error bars represent standard error of mean.

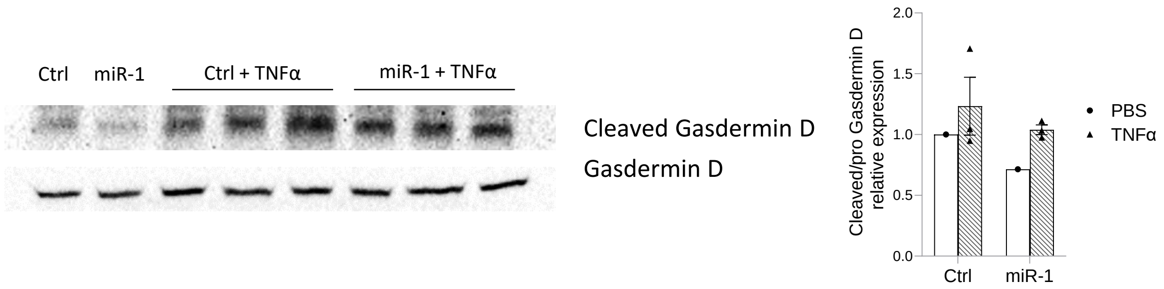

Figure S8: HPMECs were transfected and treated as in 6c. Cleaved and total Gasdermin D were detected by Western blotting. The left panel shows the immunoblot and the right panel shows the quantifications for each group normalized to the Ctrl/PBS group. Error bars represent standard error of mean.

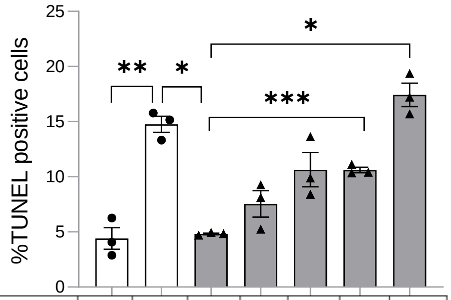

**Ctrl RNA**	+	+					
**miR-1**			+	+	+	+	+
**TNFα**		+	+	+	+	+	+
**rANGPT2 (ng/ml)**	0	0	0	200	1000	2000	4000

Figure S9: HUVECs were transfected with miR-1 (or control RNA) and treated with TNFα (10 ng/ml) 24 hours after transfection. Cells were then exposed to increasing amount of recombinant ANGPT2 (200, 1000, 2000 or 4000 ng/ml) and harvested 24 hours after TNFα treatment. Cell death was measured by TUNEL analysis. %TUNEL positive cells were determined as in 'Figure 3a' (n=3, *p<0.007, **p=0.0015, ***p=0.0009, Welch test). The graph represents data from a representative experiment out of two. Error bars represent standard error of mean.

Table S1: Genes upregulated by TNF-α and common between HUVEC, HPMEC and HDMECs (p value <0.05, fold change > 0.5

1	UBD	51	SLC7A2	101	IRAK2	151	LAMC2
2	EBI3	52	TTC39A	102	TNFAIP2	152	HLA-B
3	CXCL5	53	NFKBIE	103	NFKB1	153	BACH2
4	LTB	54	TRAF1	104	CXCL1	154	SH3RF3
5	CCL2	55	SH3BP5	105	SMAD3	155	PANX1
6	TNFRSF4	56	CSF2RB	106	VPS37C	156	BTN3A3
7	ICAM1	57	NCOA7	107	C11orf82	157	ZNF697
8	CX3CL1	58	CD74	108	TAP1	158	TRIM16
9	TNFRSF9	59	DOC2B	109	LACC1	159	MOB3C
10	BIRC3	60	PDZD2	110	SDC4	160	ST6GAL1
11	SELE	61	SLFN5	111	PARP14	161	RELT
12	CD83	62	CD69	112	CITED4		
13	CXCL11	63	C16orf46	113	TAPBP		
14	VCAM1	64	SLC12A2	114	LURAP1L		
15	RND1	65	JAK3	115	BCL3		
16	JAM2	66	LOC646999	116	APOBEC3G		
17	TNFAIP3	67	TNFSF18	117	CREB5		
18	RELB	68	CLEC1A	118	TLR3		
19	PLA2G4C	69	IKBKE	119	RASD1		
20	LRG1	70	SAMD14	120	MIR155HG		
21	S100A3	71	PMAIP1	121	CSRP2		
22	HDAC9	72	OPTN	122	SELM		
23	IL32	73	HOXB9	123	IPMK		
24	POU2F2	74	TCF7	124	GRAMD3		
25	LOC100126784	75	DDX58	125	IL18R1		
26	CSF1	76	STARD10	126	RIPK2		
27	IFIH1	77	IL8	127	ZC3H7B		
28	CLDN1	78	CBR3	128	GBP1		
29	ICOSLG	79	APLF	129	PDE5A		
30	NFKB2	80	NFE2L3	130	CYLD		
31	IL34	81	CXCL2	131	C2CD4B		
32	CBLN3	82	CNKSR3	132	JAG1		
33	KIAA1217	83	PLLP	133	JUNB		
34	DAPK2	84	CXCL3	134	ARL4C		
35	TMEM217	85	LOC283070	135	ITGAV		
36	SEMA7A	86	ZSWIM4	136	KIF13A		
37	IL7R	87	GXYLT2	137	ABCG1		
38	CXCL6	88	APOL3	138	TP53		
39	CAMK1D	89	SQSTM1	139	IRF1		
40	FAM129A	90	CXCR7	140	LYPD6		
41	STAP2	91	TNFSF15	141	BTN3A1		
42	NUAK2	92	PAPLN	142	LGALS9		
43	C8orf4	93	TANK	143	WARS		
44	SLC2A6	94	ANGPT2	144	SAMD9L		
45	MSX1	95	CTHRC1	145	SERPINE1		
46	NFKBIA	96	TNIP1	146	NFKBIB		
47	F2RL1	97	RHBDF2	147	IL3RA		
48	TIFA	98	TRAF3	148	PDCD1LG2		
49	CTSS	99	APOL6	149	BTN2A2		
50	PSMB9	100	ISG20	150	SOX7		

Table S2:Genes upregulated by TNF-α and have predicted miR-1 binding site

1	CCL2	33	RHBDF2
2	TNFRSF4	34	TRAF3
3	CX3CL1	35	IRAK2
4	TNFRSF9	36	TNFAIP2
5	SELE	37	SMAD3
6	CXCL11	38	VPS37C
7	JAM2	39	TAP1
8	TNFAIP3	40	LACC1
9	RELB	41	APOBEC3G
10	CSF1	42	CREB5
11	ICOSLG	43	TLR3
12	KIAA1217	44	CSRP2
13	DAPK2	45	IPMK
14	CXCL6	46	GRAMD3
15	CAMK1D	47	ZC3H7B
16	FAM129A	48	PDE5A
17	NUAK2	49	CYLD
18	PSMB9	50	C2CD4B
19	SLC7A2	51	ITGAV
20	SH3BP5	52	KIF13A
21	CSF2RB	53	IRF1
22	CD74	54	LYPD6
23	PDZD2	55	BTN3A1
24	SLC12A2	56	WARS
25	IKBKE	57	SAMD9L
26	OPTN	58	NFKBIB
27	HOXB9	59	LAMC2
28	TCF7	60	BACH2
29	CNKSR3	61	SH3RF3
30	SQSTM1	62	BTN3A3
31	TNFSF15	63	MOB3C
32	ANGPT2	64	ST6GAL1

Table S3. Patient characteristics*.

Characteristic	Total (N=119)
**Age** (year)	68 (± 14)
**Gender** (Males, females)	73,46
**Active Smoker, %**	19.3
**Comorbidities:**	
**Pulmonary (%):**	45
COPD	75
Asthma	13
ILD	11
Bronchiectasis	4
**Cardiac (%)**	22
**Solid tumor (%)**	27
**Hematologic Malignancy (%)**	8
**Severity:**	
mSOFA	3 (1,4)
SAPS II	32 (± 11)
SpO_2/FiO_2	310 (±100)
**Outcomes:**	
Admission to ICU (%)	67.23
Length of stay (days)	12 (6,20)
Mechanical Ventilation (%)	43
Non-invasive ventilation (%)	13
Mortality at 30 days (%)	22

COPD, Chronic Obstructive Pulmonary Disease; *ILD,* Interstitial Lung Disease; *mSOFA*, modified Sequential Organ Failure Assessment; *SAPS II,* Simplified Acute Physiology Score II; *Ang-2*, angiopoietin -2; *LOS,* Length of stay; *S/F,* Oxygen Saturation/Fraction of inspired Oxygen; *IMV,* Invasive mechanical ventilation; *NIV,* noninvasive ventilation.

* Mean ± standard deviation (SD) was used to express the normal distribution data, and median, interquartile range (IQR, 25-75 percentile) was used to express the skewed data.

Table S4: Correlations of miR-1 ($2^{-\Delta\Delta CT}$)* with clinical and physiological variables in patients admitted with bacterial pneumonia (n = 119)**.

Variable	R coefficient	P value
Age	0.06386	0.4902
Male gender	0.008540	0.9266
Presence of pulmonary co-morbidities	-0.01870	0.8400
ANGPT2	-0.2088	0.0227
Bicarbonate	-0.01339	0.8851
Platelets	0.09260	0.3186
BUN	-0.04540	0.6239
WBC	-0.08151	0.3782
GFR	-0.004662	0.9599
Lactate	-0.1168	0.4447
mSOFA	-0.1444	0.1170
SAPS II	0.03610	0.6967
S/F Ratio	0.1218	0.1872
LOS***	-0.2114	0.0357
Circulatory Shock	-0.05683	0.5393
Mechanical ventilation***	-0.01768	0.8621
Days on Mechanical ventilator***	-0.08637	0.3953
30-day-mortality***	-0.1613	0.1108
Renal Replacement Therapy***	-0.1537	0.1288

ANGPT2*,* angiopoietin -2, *BUN,* Blood Urea Nitrogen, *WBC,* White blood cell, *GFR,* Glomerular filtration rate, *mSOFA*, Modified *s*equential organ failure assessment, *SAPS II*, Simplified Acute Physiology Score II, *LOS,* Length of stay, *S/F, s*aturation/Fraction of inspired Oxygen.

* MiR-1/18S was measured by qRT-PCR and normalized to the median of the whole group (2-ΔΔct) and Log transformed for this correlation analysis.

**P values are based on Pearson’s test for parametric data and Spearman's test for non-parametric data. P values not adjusted for multiple testing since this is an exploratory analysis on a heterogenous population of patients.

***Patients with code status of Do Not Intubate and Do Not Resuscitate (DNR/DNI) were removed from analysis of clinical outcomes and therefore sample size for those variables was reduced to 99.

Table S5. Patient characteristics of patients admitted to the ICU*.

Characteristic	Total (N=67)
**Age** (year)	65 (± 13)
**Gender** (Males, females)	73,46
**Severity:**	
mSOFA	3 (2,5)
SAPS II	34 (± 10)
SpO_2/FiO_2	257 (±84)
**Outcomes:**	
Length of stay (days)	15 (9,25)
Mechanical Ventilation (%)	76
Non-invasive ventilation (%)	23
Mortality at 30 days (%)	31

COPD, Chronic Obstructive Pulmonary Disease; *ILD,* Interstitial Lung Disease; *mSOFA*, modified Sequential Organ Failure Assessment; *SAPS II,* Simplified Acute Physiology Score II; *Ang-2*, angiopoietin -2; *LOS,* Length of stay; *S/F,* Oxygen Saturation/Fraction of inspired Oxygen; *IMV,* Invasive mechanical ventilation; *NIV,* noninvasive ventilation.

*Mean ± standard deviation (SD) was used to express the normal distribution data, and median, interquartile range (IQR, 25-75 percentile) was used to express the skewed data. ICU cohort consisted of patients requiring non-invasive or invasive mechanical ventilation.
